# RUNX represses *Pmp22* to drive neurofibromagenesis

**DOI:** 10.1126/sciadv.aau8389

**Published:** 2019-04-24

**Authors:** Ashley Hall, Kwangmin Choi, Wei Liu, Jonathan Rose, Chuntao Zhao, Yanan Yu, Youjin Na, Yuqi Cai, Robert A. Coover, Yi Lin, Eva Dombi, MiOk Kim, Ditsa Levanon, Yoram Groner, Elisa Boscolo, Dao Pan, P. Paul Liu, Q. Richard Lu, Nancy Ratner, Gang Huang, Jianqiang Wu

**Affiliations:** 1 Cincinnati Children’s Hospital Medical Center, Division of Experimental Hematology and Cancer Biology, Cancer and Blood Diseases Institute, University of Cincinnati, 3333 Burnet Ave., Cincinnati, OH 45229, USA.; 2 Department of Cancer and Cell Biology, College of Medicine, University of Cincinnati, Cincinnati, OH 45267, USA.; 3 Pediatric Oncology Branch, National Cancer Institute, Bethesda, MD 20892, USA.; 4 Department of Epidemiology and Biostatistics, UCSF, Box 0128, 1450 3rd St. Suite 285, San Francisco, CA 94143, USA.; 5 Department of Molecular Genetics, Weizmann Institute of Science, Rehovot, Israel.; 6 Department of Pediatrics, University of Cincinnati College of Medicine, Cincinnati, OH 45267, USA.; 7 National Human Genome Research Institute, National Institutes of Health, Bethesda, MD 20892, USA.

## Abstract

Patients with neurofibromatosis type 1 (NF1) are predisposed to develop neurofibromas, but the underlying molecular mechanisms of neurofibromagenesis are not fully understood. We showed dual genetic deletion of *Runx1* and *Runx3* in Schwann cells (SCs) and SC precursors delayed neurofibromagenesis and prolonged mouse survival. We identified peripheral myelin protein 22 (*Pmp22/Gas3*) related to neurofibroma initiation. Knockdown of *Pmp22* with short hairpin RNAs increased *Runx1^fl/fl^;Runx3^fl/fl^;Nf1^fl/fl^;DhhCre* tumor-derived sphere numbers and enabled significantly more neurofibroma-like microlesions on transplantation. Conversely, overexpression of *Pmp22* in mouse neurofibroma SCs decreased cell proliferation. Mechanistically, RUNX1/3 regulated alternative promoter usage and induced levels of protein expression of *Pmp22* to control SC growth. Last, pharmacological inhibition of RUNX/core-binding factor β (CBFB) activity significantly reduced neurofibroma volume in vivo. Thus, we identified a signaling pathway involving RUNX1/3 suppression of *Pmp22* in neurofibroma initiation and/or maintenance. Targeting disruption of RUNX/CBFB interaction might provide a novel therapy for patients with neurofibroma.

## Teaser

RUNX 1 and RUNX3 drive *Nf1* neurofibromagenesis by mediating the promoter usage and inducing levels of protein expression of PMP22.

## INTRODUCTION

Neurofibromatosis type 1 (NF1) is a common inherited human disorder, with a frequency of 1:2500 worldwide (*1*). About 95% of patients with NF1 develop dermal and/or plexiform neurofibromas, for which no effective cure exists. Often, surgery removal is impossible because of the integration of tumor into critical peripheral nerves (*1*). The cytostatic MAPK (mitogen-activated protein kinase) kinase inhibitor selumetinib has shown promising efficacy, but tumors regrow after stopping the treatment (*2*, *3*). New therapeutic strategies and targets for the treatment of neurofibromas are urgently needed.

The drivers of neurofibromagenesis have not been fully identified. *NF1* encodes a RAS guanosine triphosphatase–activating protein that activates downstream RAS pathways. Therefore, loss of *NF1* is considered a potential major driver of neurofibromagenesis (*4*, *5*). Other genes, such as *STAT3* and *EGFR*, also contribute to neurofibroma formation (*6*, *7*). However, none of them can eliminate tumor formation, suggesting that neurofibroma formation involves multiple drivers.

Three RUNT-related transcription factor (RUNX1, RUNX2, and RUNX3) genes are present in human and in mouse. Their gene products share many structural similarities but have distinct biological activities. RUNX1 (also called AML1) is required for the maturation of megakaryocytes and differentiation of T and B cells. RUNX2 is critical for skeletal morphogenesis (*8*). RUNX3 is important for neurogenesis of proprioceptive neurons in the dorsal root ganglia (DRG) and for hematopoiesis (*9*, *10*). All three RUNX proteins bind to the core-binding factor β (CBFB) via the same protein motif. CBFB lacks a DNA binding domain but, when bound to RUNX, substantially increases the Runt domain DNA binding affinity, thereby enhancing RUNX transcriptional activities (*11*). RUNX can function as either tumor suppressors or oncogenes. RUNX1 has been implicated as a tumor suppressor in solid tumors, including breast cancer, esophageal adenocarcinoma, colon cancer, and possibly, prostate cancer and as an oncogene in skin cancer, endometrial cancer, and epithelial cancer (*12*). RUNX2 has been implicated in metastasis including bone metastasis. RUNX3 acts as a tumor suppressor in gastric cancer but functions as an oncogene in ovarian cancers (*12*). Extracellular signal–regulated kinase (ERK) signaling (downstream of NF1/RAS/MAPK) phosphorylates RUNX1 at S276/S293 or arginine methylation of R206 and R210 (*13*). ERK phosphorylation caused by loss of NF1 increases RUNX1 activity and contributes to neurofibroma formation (*14*).

Schwann cells (SCs) develop from neural crests, and *Nf1^−/−^* SCs and/or their precursors are cells of origin for neurofibromas (*15*, *16*). There are both myelinating and nonmyelinating SCs within a neurofibroma, and neurofibromas harbor increased Remak bundle–associated (nonmyelinating) SCs. One of the SC’s functions is to form a myelin sheath containing the peripheral myelin protein 22 (PMP22). *PMP22* is mainly expressed in myelinating SCs (*17*, *18*) and neurons (*19*). Its expression is regulated via transcriptional regulation and posttranscriptional modification. *Pmp22* has two major different mRNAs that differ only in their 5′-untranslated regions (5′-UTRs). This difference causes alternative usage of two promoters located upstream of the exons 1A (P1) and 1B (P2). Both P1 and P2 are developmentally regulated in SCs and contribute to Pmp22 levels in mature SCs. P1 is SC specific, and P2 is more often used in other tissues where *Pmp22* is expressed at a lower level (*17*, *18*). Increased PMP22 in gene dosage results in the inherited demyelinating peripheral neuropathies Charcot-Marie-Tooth disease type 1A (*20*), while decreases cause hereditary neuropathy with liability to pressure palsies (*21*).

The PMP22 gene expression has been detected within and outside the nervous system and in several cancers, and a number of studies have reported either up- or down-regulation of the gene/protein in different models. Amplification of chromosome 17p11.2 region and the PMP22 protein expression have been associated with human osteosarcoma and glioblastoma (*22*). The PMP22 gene/protein expression is markedly up-regulated in gastric cancer–derived tumorspheric cells and contributes to cell proliferation, tumorsphere formation, and chemoresistance to cisplatin (*23*). Conversely, the *PMP22* gene expression is diminished in metastatic carcinoma cells compared with primary carcinoma cells, suggesting that it might serve as a prognostic marker (*24*). Here, we showed that dual deletion of mouse *Runx1/3* in SCs and SC precursors (SCPs) significantly delayed neurofibromagenesis and prolonged mouse survival. We showed that RUNX1/3 regulated *Pmp22* expression by switching alternative promoter usage and markedly induced levels of protein expression of *Pmp22* to drive neurofibromagenesis. We also showed that pharmacological inhibition of RUNX/CBFB activity significantly reduced mouse neurofibroma growth in vivo, implicating a novel signaling pathway involving RUNX1/3 repression of *Pmp22* in neurofibroma initiation and/or maintenance.

## RESULTS

### Conditional knockout of *Runx1* transiently delays neurofibroma growth and induces compensatory overexpression of *Runx3* in the *Runx1*^*fl/fll*^*;Nf1*^*fl/fl*^*;DhhCre* mouse model

We have previously shown that targeted genetic deletion of *Runx1* in SCs and SCPs decreases neurofibroma formation at 4 months (*14*), but we found that there was no difference in tumor volume at 7 months of age (*P* = 0.38) (fig. S1A). Because all three RUNX proteins (with CBFB) bind to the same DNA motif to exert their effects, it is possible that phenotypes observed upon conditional inactivation of *Runx1* were attenuated by compensation of *Runx2* and/or *Runx3*. Quantitative reverse transcription polymerase chain reaction (qRT-PCR) on *Runx1^fl/fl^;Nf1^fl/fl^;DhhCre* mouse tumors/DRGs and age-matched *Nf1^fl/fl^;DhhCre* tumors indicated that *Runx3* expression displayed a pronounced time-dependent, increase (fig. S1B). Immunohistochemistry (IHC) on 7-month-old *Runx1^fl/fl^;Nf1^fl/fl^;DhhCre* mouse DRG/tumors verified stronger *Runx3* expression compared with age-matched *Nf1^fl/fl^;DhhCre* tumors (fig. S1C), suggesting induced compensation of *Runx3* upon conditional knockout of *Runx1*.

To determine whether *Runx3* affects *Runx1* knockout SCPs, we used *shRunx3* to transduce *Runx1^fl/fll^;Nf1^fl/fl^;DhhCre* DRG/tumor-derived mouse neurofibroma spheres. We found a significant decrease in the numbers of neurofibroma spheres in all three tested clones compared with shnon-target control (*shNT*; fig. S1D), indicating that Runx3 compensated for the tumor growth upon conditional inactivation of *Runx1* in the *Nf1^fl/fl^;DhhCre* cells.

### Dual deletion of *Runx1/3* prolongs mouse survival and decreases tumor number and volume in the *Nf1*^*fl/fl*^*;DhhCre* neurofibroma mouse model

**Fig. 1:**
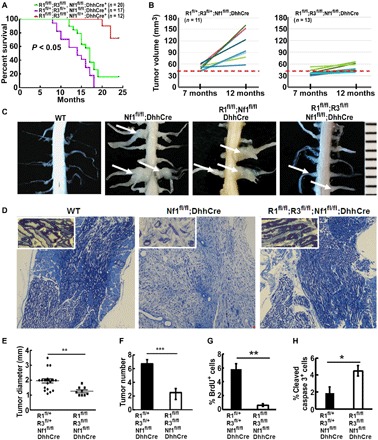
Dual deletion of Runx1/3 prolongs survival time and decreases tumor number and size in the *Nf1^fl/fl^;DhhCre* neurofibroma mouse. (**A**) Kaplan-Meier survival curve. Purple, *Runx1^fll+^;Runx3 ^fl/+^;Nf1^fllfl^;DhhCre;* green, Runx1*^fl/fl^;Runx3^fl/fl^;Nf1^fl/fl^;DhhCre;* red, Runx1*^fl/fl^;Runx3^fl/fl^;Nf1^fl+^*;*DhhCre*. (**B**) Each mouse volume at 7 and 12 months in controls (*Runx1^fll+^;Runx3 ^fl/+^;Nf1^fllfl^;DhhCre* mice) (left) and Runx1*^fl/fl^;Runx3^fl/fl^;Nf1^fl/fl^;DhhCre* mice (right). Dashed lines indicate all the control mice volume >40 mm^3^ at 7 months, while the double-knockout mice volume varied. Mouse tumor volume statistical analysis: Mixed model analysis. Mixed-effects analysis of tumor volume showed *P* < 0.001 between *Runx1^fll+^;Runx3 ^fl/+^;Nf1^fllfl^;DhhCre* mice and Runx1*^fl/fl^;Runx3^fl/fl^;Nf1^fl/fl^;DhhCre* mice at 7 and 12 months, respectively. (**C**) Representative gross dissections of thoracic paraspinal neurofibromas and nerve roots in 12 months of age-matched WT (left), *Nf1^fl/fl^*;*DhhCre* mice (second from the left), Runx1*^fl/fl^;Nf1^fl/fl^;DhhCre* mice (third from the left), and *Runx1^fl/fl^;Runx3^fl/fl^;Nf1^fl/fl^;DhhCre* mice (right). White arrows point to tumors. Ruler showed 1-mm markings. (Photo credit: Ashley Hall and Jianqiang Wu). (**D**) Representative photographs of toluidine blue–stained semithin sections from age-matched WT (left), *Nf1^fl/fl^*;*DhhCre* (middle), and *Runx1^fl/fl^;Runx3^fl/fl^;Nf1^fl/fl^;DhhCre* (right) mouse DRGs. Top-left corner insertions are high magnification of imagings to each corresponding genotype. (**E**) Tumor diameter in the *Runx1^fll+^;Runx3 ^fl/+^;Nf1^fllfl^;DhhCre* mice (circles; *n* = 3 mice with 20 tumors) and Runx1*^fl/fl^;Runx3^fl/fl^;Nf1^fl/fl^;DhhCre* mice (squares; *n* = 4 mice with nine tumors) at 12 months. (**F**) Average tumor number per mouse at 12 months in the *Runx1^fll+^;Runx3^fl/+^;Nf1^fllfl^;DhhCre* mice (black bar; *n* = 3) and Runx1*^fl/fl^;Runx3^fl/fl^;Nf1^fl/fl^;DhhCre* mice (white bar; *n* = 4). (**G**) Cell proliferation shown as percentage of BrdU^+^ cells in *Runx1^fll+^;Runx3^fl/+^;Nf1^fllfl^;DhhCre* mice (black bar; *n* = 5) and Runx1*^fl/fl^;Runx3^fl/fl^;Nf1^fl/fl^;DhhCre* mice (white bar; *n* = 5). (**H**) Cell death shown as percentage of CC3^+^ cells in *Runx1^fll+^;Runx3^fl/+^;Nf1^fllfl^;DhhCre* mice (black bar; *n* = 5) and Runx1*^fl/fl^;Runx3^fl/fl^;Nf1^fl/fl^;DhhCre* mice (white bar; *n* = 5). (D to G) Unpaired Student’s *t* test. *P < 0.05, **P < 0.01, and ***P < 0.001. *R1^fl/fl^;R3^fl/fl^;Nf1^fl/fl^;DhhCre* = *Runx1^fl/fl^;Runx3^fl/fl^;Nf1^fl/fl^;DhhCre*.

To test whether *Runx1/3* cooperates to drive neurofibromagenesis, we carried out survival analysis. Kaplan-Meier analysis revealed a significant survival difference between *Runx1^fl/fl^;Runx3^fl/fl^;Nf1^fl/fl^;DhhCre* mice and littermate *Runx1^fl/+^;Runx3^fl/+^;Nf1^fl/fl^;DhhCre* mice (*P* < 0.05) (Fig. 1A). We could not obtain littermate *Runx^WT^;Runx3^WT^;Nf1^fl/fl^;DhhCre* mice because of the limitation of the breeding strategy, but we did detect significantly longer survival time when we compared the *Runx1^fl/fl^;Runx3^fl/fl^;Nf1^fl/fl^;DhhCre* mice with previously published cohorts of *Runx^WT^;Runx3^WT^;Nf1^fl/fl^;DhhCre* mice that harbored similar background. No significance was detected on survival time between *Runx^WT^;Runx3^WT^;Nf1^fl/fl^;DhhCre* and *Runx1^fl/+^;Runx3^fl/+^;Nf1^fl/fl^;DhhCre* mice, suggesting that loss of each allele of Runx1 and Runx3 only might not change tumor penetration rate. We also quantified total neurofibroma burden by volumetric measurement of magnetic resonance imaging (MRI) scans, followed by mixed-effects analysis of tumor volume. Tumor size was significantly smaller at 7 and 12 months in *Runx1^fl/fl^;Runx3^fl/fl^;Nf1^fl/fl^;DhhCre* mice (*n* = 13) compared with littermate controls (*Runx1^fl/+^;Runx3^fl/+^;Nf1^fl/fl^;DhhCre*; *n* = 11; Fig. 1B).

In the *Nf1^fl/fl^;DhhCre* model, each mouse develops 4 to 20 neurofibromas (*16*). We hypothesized that if Runx1/3 contributes to neurofibroma initiation, then tumor number should be reduced in *Runx1^fl/fl^;Runx3^fl/fl^;Nf1^fl/fl^;DhhCre* mice. Gross dissections of 12-month-old mice showed that *Runx1^fl/fl^;Runx3^fl/fl^;Nf1^fl/fl^;DhhCre* mice had significantly fewer spinal cord tumors/mouse versus age-matched *Runx1^fl/+^;Runx3^fl/+^;Nf1^fl/fl^;DhhCre* littermates at spinal cords (Fig. 1, C and E). Consistent with volumetric MRI scan results, neurofibroma diameter measured on spinal root–dissected sections was also significantly smaller in *Runx1^fl/fl^;Runx3^fl/fl^;Nf1^fl/fl^;DhhCre* mice versus *Runx1^fl/+^;Runx3^fl/+^;Nf1^fl/fl^;DhhCre* mice at 12 months of age (Fig. 1, C and F). Hematoxylin and eosin (H&E) staining showed that all tumors were still genetically engineered mouse–grade 1 neurofibroma (not shown). On toluidine blue–stained semithin sections, *Runx1^fl/fl^;Runx3^fl/fl^;Nf1^fl/fl^;DhhCre* mouse DRG showed that most of the myelin sheaths were restored to wild-type (WT) levels, with some abnormal thicker myelin sheaths. On the other hand, age-matched *Nf1^fl/fl^;DhhCre* mouse DRG had few small myelin sheaths (Fig. 1D). 5-bromo-2′-deoxyuridine–positive (BrdU^+^)–proliferating cell numbers in neurofibroma tissue sections significantly decreased in *Runx1^fl/fl^;Runx3^fl/fl^;Nf1^fl/fl^;DhhCre* neurofibromas compared with *Runx1^fl/+^;Runx3^fl/+^;Nf1^fl/fl^;DhhCre* mouse tumors (Fig. 1G). The numbers of apoptotic cells (cleaved caspase 3, CC3^+^) were slightly increased (Fig. 1H). Therefore, glial cell *Runx1/3* regulates *Nf1*-deficient tumor cell proliferation in neurofibromas, and coactivation of *Runx1/3* in SCs and/or SCPs is important for neurofibroma initiation and growth.

### *Runx1/3* drives neurofibromagenesis by activating oncogenic pathways and reprogramming the neuronal and immune systems

We performed RNA sequencing (RNA-seq) to investigate whether *Runx1/3* mediates changes in gene regulation involved in neurofibromagenesis. To avoid the complexity of heterozygous knockout of *Runx1/3*, we compared RNA-seq gene expression profiles of *Nf1^fl/fl^;DhhCre* tumors and *Runx1^fl/fl^;Runx3^fl/fl^;Nf1^fl/fl^;DhhCre* mouse DRG/tumors using a false discovery rate (FDR) *P* value of <0.05 and >2-fold change as the significant threshold. We identified 2887 differentially expressed RUNX1/3-responsive genes, of which 1677 genes were up-regulated and 1210 genes were down-regulated in *Runx1^fl/fl^;Runx3^fl/fl^;Nf1^fl/fl^;DhhCre* DRG/tumors compared with *Nf1^fl/fl^;DhhCre* tumors (fig. S2A and table S1). Gene set enrichment analysis showed increased neuronal system [normalized enrichment score (NES) = 0.472, *P* = 0.001], decreased cytokine signaling (NES = −0.347, *P* = 0.004), extracellular matrix organization (NES = −0.405, *P* = 0.004), and innate immune system (NES = −0.281, *P* = 0.015) (fig. S2B). Gene ontology pathway analysis showed similar deregulation of immune-related pattering (fig. S2C). In contrast, extensive activation of pathways associated with SC development and myelination or apoptosis, including neuronal system, axon guidance, diacylglycerol (DAG). and inositol 1,4,5-trisphosphate signaling, oxidative phosphorylation, and ion channel transportation, was remarkably up-regulated (fig. S2D).

### Combined transcriptome profiling, ChIP-seq, and ATAC-seq analysis identifies 14 highly direct targets of RUNX1/3

**Fig. 2:**
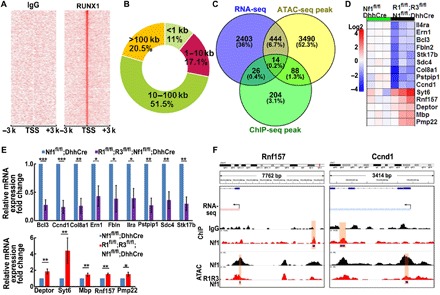
Combined transcriptome profiling, ChIP-seq, and ATAC-seq analysis identifies 14 highly plausible direct targets of Runx. (**A**) Heat maps showed the antibody-binding pattern around the TSSs region. Each panel represents 3 kb upstream and downstream of the TSSs. Left, IgG control; right, RUNX1 antibody. (**B**) Circle chart depicted RUNX1-binding site distribution in relation to the nearest annotated TSSs. Numbers represented distance from TSS (kb), and percentages represented percentage of bound regions. (**C**) Venn diagram showed the overlap of genes among RNA-seq DEGs, ChIP-seq peak, and ATAC-seq peak. For ChIP-seq and ATAC-seq, we consider differential peaks detected within ±20 kb around TSS. (**D**) Heat map showed the 14 commonly shared genes from *Nf1^fl/fl^;DhhCre* and Runx1*^fl/fl^;Runx3^fl/fl^;Nf1^fl/fl^;DhhCre* tumor RNA-seq. (**E**) qRT-PCR confirmed the relative mRNA expression of the 14 targets. (**F**) Representative two genes (Rnf157: up-regulated gene; Ccnd1: down-regulated gene) showed their RNA-seq gene expression (blue: down-regulation; red: up-regulation), RUNX1-binding ChiP-seq peak, and ATAC-seq peak. *P < 0.05, **P < 0.01.

To identify the genes that are directly regulated by Runx, we performed chromatin immunoprecipitation sequencing (ChIP-seq) to assess the genome-wide occupancy of RUNX1 in *Nf1*-deficient tumor cells. Eight hundred and seventy-seven differential peaks [RUNX1 versus immunoglobulin G (IgG)] were predicted using HOMER (FDR < 0.001). RUNX1 peak density was enriched within elements ±3 kb proximal to the transcriptional start sites (TSSs), and a large proportion of RUNX1 peaks were located in enhancer regions (Fig. 2A and fig. S3A). We detected a small fraction of peaks (11%) close to the ±1-kb TSS; most of the peaks were located in intergenic regions beyond 1 kb from the nearest TSS (Fig. 2B). Most RUNX1-bound regions (79.5% of RUNX1 ChIP-seq peaks) were located <100 kb from known TSS, and only 20.5% were found in >100 kb from TSS (Fig. 2B). De novo transcriptional factor (TF) enrichment results showed that RUNX(Runt)/HPC7-Runx1 (*P* = 1 × 10^−533^, consensus = NNKYTGTGGTTW) and Fra1(bZIP)/BT549-Ra1 (*P* = 1 × 10^−134^, consensus = NRTGASTCAB) were the most enriched TF motifs (fig. S3B).

RUNX mediates changes in chromatin structure (*25*). We performed assay for transposase-accessible chromatin sequencing (ATAC-seq) to determine whether RUNX is associated with changes in chromatin accessibility in our system. To avoid the complexity of cell type heterogeneity within a tumor, we FACS (fluorescence-activated cell sorting)–sorted enhanced green fluorescent protein–positive (EGFP^+^) SC cells from DRG/tumor of *Runx1^fl/fl^;Runx3^fl/fl^;Nf1^fl/fl^;DhhCre* and *Nf1^fl/fl^;DhhCre* mice, where EGFP serves as a reporter in the SC lineage and represents *Runx1^−/−^;Runx3^−/−^;Nf1^−/−^* and *Runx1^WT^;Runx3^WT^;Nf1^−/−^* SCs, respectively. We identified 6979 differential ATAC-seq peaks in total (fig. S3C). Kyoto Encyclopedia of Genes and Genomes pathway by sorting of differential ATAC-seq peaks revealed areas of significances as highly enriched in protein processing in endoplasmic reticulum (ER) (log *P* = −18.4), epidermal growth factor receptor (EGFR) tyrosine kinase inhibitor resistance (log *P* = −14.1), MAPK signaling (log *P* = −13.0), transforming growth factor–β (TGF-β) signaling (log *P* = −12.0), Ras signaling (log *P* = −11.7), mammalian target of rapamycin signaling (log *P* = −10.9), phosphatidylinositol 3-kinase–Akt signaling (log *P* = −9.6), and Wnt signaling (log *P* = −8.5) (fig. S3D).

By cross-comparing ChIP-seq targets (±20 kb from TSS and *P* < 0.01), ATAC-seq open chromatin genes (±20 kb from TSS), and RNA-seq differentially expressed genes (DEGs) (FDR *P* < 0.05 and >2-fold change), we identified 14 genes that were highly plausible direct targets of RUNX1/3 (Fig. 2, C and D). The up-regulated genes were related to SC myelination (*Mbp* and *Pmp22*), kinase activity (*Deptor*), E3 ubiquitin ligase (*Rnf157*), and mediation of Ca^2+^ regulation of exocytosis (*Syt6*). The down-regulated genes were related to oncogenes that activate nuclear factor κB signaling (*Bcl3* and *Stk17b*), cell cycle (*Ccnd1*), ER stress (*Ern1*), cell surface interacting gene (*Sdc4*), promotion of T helper 2 cell differentiation gene (*Il4ra*), and cell structure maintenance genes (*Col8a1*, *Fbln2*, and *Pstpip1*) (Fig. 2D). The relative mRNA expression of these genes was confirmed by qRT-PCR (Fig. 2E). Gene expression data were consistently correlated with chromatin accessibility (Fig. 2F).

### *Pmp22* is a direct target of RUNX1/3 in neurofibroma

**Fig. 3:**
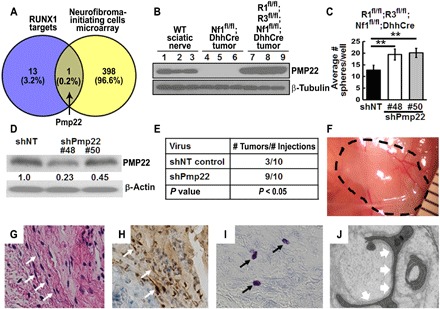
Loss of *Nf1* mediated PMP22 functions as tumor suppressor to contribute to neurofibroma initiation. (**A**) Venn diagram showed the overlap of the 14 RUNX1/3 target genes with human neurofibroma-initiating cell microarray DEGs. (**B**) Western blot of PMP22 in WT mouse sciatic nerves (1 to 3), *Nf1^fl/fl^;DhhCre* tumors (4 to 6), and *Runx1^fl/fl^;Runx3^fl/fl^;Nf1^fl/fl^;DhhCre* tumors (7 to 9). β-Tubulin was used as a loading control. (**C**) *ShPmp22* partially rescued numbers *of Runx1^fl/fl^;Runx3^fl/fl^;Nf1^fl/fl^;DhhCre* spheres. **P < 0.01. (**D**) Western blot showed the levels of *Pmp22* knockdown by *shPmp22*. Numbers indicated the percentage of knockdown. (**E**) Neurofibroma-like lesions formed after subcutaneous injection of *shNT*- or *shPmp22*-transduced *Runx1^fl/fl^;Runx3^fl/fl^;Nf1^fl/fl^;DhhCre* DRG/neurofibroma-derived sphere cells. Statistics: Fisher’s exact test. *P* < 0.05. (**F**) A representative gross photograph of a lesion (dashed black circle) under reflected skin in a *nu/nu* mouse injected with *shPmp22*-transduced *Runx1^fl/fl^;Runx3^fl/fl^;Nf1^fl/fl^;DhhCre* DRG/neurofibroma-derived sphere cells. Ruler showed 1-mm markings. (Photo credit: Ashley Hall and Jianqiang Wu). (**G**) H&E-stained section of the tumor from (G) showed spindle cells (white arrows). (**H**) IHC showing S100β^+^ cells (brown; white arrows) in tumor. Blue is H&E counterstain. (**I**) Toluidine blue staining showed mast cells (black arrows). (**J**) Electronic micrograph showed lesions containing SCs, identified by continuous basal lamina in high magnification (white arrows).

We previously showed that P75^+^/EGFR^+^ SCs are potential neurofibroma-initiating cells (*26*) and that they showed differential gene expression profile compared with WT P75^+^/EGFR^−^ SCs. To identify genes that might be related to neurofibroma initiation, we compared these 14 genes to those overexpressed in a neurofibroma tumor–initiating cell microarray dataset GSE122773 (*14*). We identified *Pmp22* as the only common gene (Fig. 3A). The *Pmp22* expression was confirmed by qRT-PCR (Fig. 2E). Western blots showed that PMP22 protein was expressed in WT mouse sciatic nerves but was almost undetectable in *Nf1^fl/fl^;DhhCre* mouse neurofibromas, while robust expression in *Runx1^fl/fl^;Runx3^fl/fl^;Nf1^fl/fl^;DhhCre* mouse DRG/tumors was observed (Fig. 3B). We analyzed the existing transcriptomic data (www.ncbi.nlm.nih.gov/geo/query/acc.cgi?acc=GSE41747 for mouse and www.ncbi.nlm.nih.gov/geo/query/acc.cgi?acc=GSE14038 for human). We found decreased gene expression on the SC differentiation/myelination markers (*PMP22*, *MPZ*, and *MBP*) in both mouse and human plexiform neurofibromas versus WT nerve controls. On the contrary, RUNX family gene (*RUNX1*, *RUNX2*, and *RUNX3*) expression was increased in plexiform neurofibromas (fig. S4, A and B), suggesting that RUNX factors might drive the dedifferentiation/myelination of SCs in our model.

Reports show that *Pmp22* is also regulated by other genes such as Sox10, Egr2, and Hippo regulators (*27*). To determine whether these regulators are related to Pmp22 expression in our system, we searched the gene expression change on the *Runx1^fl/fl^;Runx3^fl/fl^;Nf1^fl/fl^;DhhCre* versus *Nf1^fl/fl^;DhhCre* tumor RNA-seq data. We found that there was no significant differential expression among these genes (fig. S5), suggesting that more sample replicates might be needed or these regulators might be less relevant to *Pmp22* expression upon loss of *Nf1*.

### Loss of *Nf1* mediated PMP22 functions as tumor suppressor to contribute to neurofibroma initiation

We used neurofibroma sphere culture, a system enabling detection of growth and self-renewal of nervous system stem cells/progenitors, to determine whether *Pmp22* was related to SCP growth upon loss of *Nf1*. We knocked down *Pmp22* from *Runx1^fl/fl^;Runx3^fl/fl^;Nf1^fl/fl^;DhhCre* mouse DRG/neurofibroma-derived spheres, which has high *Pmp22* expression using *shPmp22*s or shNT. Sphere numbers were significantly increased 4 days after *shPmp22* transduction versus *shNT* controls (Fig. 3D). The overall average sphere size was similar (not shown). We confirmed decreased *Pmp22* by Western blot (Fig. 3E). Thus, loss of Runx1/3 increased Pmp22 expression in *Runx1^fl/fl^;Runx3^fl/fl^;Nf1^fl/fl^;DhhCre*, while decreasing *Pmp22* expression increased *Runx1^fl/fl^;Runx3^fl/fl^;Nf1^fl/fl^;DhhCre* SCP’s self-renewal in vitro.

In many cancers, self-renewing stem/progenitor-like cells contribute to tumorigenesis. To test whether tumor formation was affected by *Pmp22* reduction, we transplanted the above dissociated *shPmp22* or *shNT* control sphere cells into nude mice (*nu/nu*). Eight weeks after transplantation, neurofibroma-like microlesions were detected in 8 of 10 *nu/nu* mice, which were transplanted subcutaneously with *shPmp22* lentivirus-transduced *Runx1^fl/fl^;Runx3^fl/fl^;Nf1^fl/fl^;DhhCre* mouse DRG/tumor-derived sphere cells, while fewer lesions (3 of 10) were detected in *shNT* controls (*P* < 0.05) (Fig. 3, E and F). H&E staining of tissue sections showed features of neurofibroma-like lesion with spindle shape cells (Fig. 3G), anti-S100β^+^ SCs (Fig. 3H), and toluidine blue–positive mast cells (Fig. 3I). On electron micrographs, these lesions contained SCs, identified by their wrapping of axons and continuous basal lamina (Fig. 3J). These in vitro and in vivo genetic results supported the conclusion that *Pmp22* was an important target of *Runx1/3*-mediated neurofibromagenesis.

### Overexpression of *Pmp22* in mouse neurofibroma SCs decreases cell proliferation

**Fig. 4:**
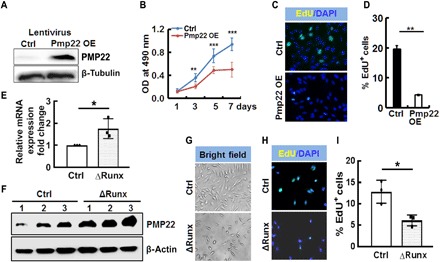
*Pmp22* inhibits mouse neurofibroma SC proliferation, and overexpression of *Pmp22* induces cell senescence. (**A**) Western blot showing PMP22 expression on lentivirus control (Ctrl) or PMP22 overexpression (OE) lentivirus-transduced mouse neurofibroma SCs. β-Tubulin was used as a loading control. (**B**) MTS assay showing significantly decreased cell growth in Pmp22 overexpressing mouse neurofibroma SCs compared with lentivirus control cells starting at day 3 and stopping cell growth at day 5. OD, optical density. (**C**) Representative EdU (green) and 4′,6-diamidino-2-phenylindole (DAPI) (blue) images on control (top) and Pmp22 overexpressing (bottom) mouse neurofibroma SCs. (**D**) Quantification of percentage of EdU^+^ cells on control (black bar) and Pmp22 overexpressing cells (white bar). (**E**) qRT-PCR of the relative mRNA expression of Pmp22 on Δ*Runx* cells normalized to control (*n* = 3 per group). (**F**) Representative Western blot of Pmp22 on three independent controls and three independent ΔRunx cells. (**G**) Representative bright-field images of mouse neurofibroma SCs. Top (control): Cells were transduced by lentivirus, but the RUNX-*Pmp22*–binding sites remained intact. Bottom (Δ*Runx*-*Pmp22*): Homozygous deletion of five putative RUNX-binding sites in *Pmp22* gene by CRISPR-Cas9 approach (Δ*Runx*). (**H**) Representative EdU (green) and DAPI (blue) images on control (top) and Δ*Runx* (bottom) mouse neurofibroma SCs. (**I**) Quantification of percentage of EdU^+^ cells on control (white bar; *n* = 3) and Δ*Runx* cells (light gray bar; *n* = 3). *P < 0.05, **P < 0.01, and ***P < 0.001.

We overexpressed *Pmp22* in mouse neurofibroma SCs to further determine the role of *Pmp22* on SC growth. Western blot confirmed *Pmp22* expression on these selected cells (Fig. 4A). MTS assay showed that cell growth decreased significantly by passage 4 cells (day 3), and almost no cell growth was detected from days 5 to 7 (Fig. 4B). 5-ethynyl-2′-deoxyuridine (EdU) assays showed significantly decreased cell proliferation (21 days after puromycin selection) (Fig.4, C and D) but no difference in apoptosis as detected by CC3.

### Deletion of RUNX-binding sites in *Pmp22* gene decreases cell proliferation and increases *Pmp22* expression

ChIP-seq identified five putative RUNX-binding sites (TGTGG) in the *Pmp22* gene (fig. S6A). To determine the importance of these RUNX-*Pmp22*–binding sites, we used a CRISPR-Cas9 approach to delete this sequence in mouse neurofibroma SCs. We identified four independent homozygous deletion clones (clones #12, #65, #79, and #85; designated Δ*Runx*). We confirmed the deletion by sequencing (fig. S6, B and C). Consistent with the in vivo genetic results, qRT-PCR showed increased expression of PMP22 at both RNA and protein levels on the analyzed homozygous clones compared with controls (Fig. 4, E and F). We noted that the in vitro Pmp22 RNA expression change was similar to the genetic data (~2-folds), but the induction of protein levels was much less marked compared with the in vivo genetic data, and we also detected PMP22 protein expression in WT controls in Western blot. One of the possible explanations is that these cells have been cultured in vitro for a long time (~4 months) in the presence of forskolin, which can increase both SC PMP22 mRNA and protein expression in vitro. The Δ*Runx* cells showed decreased cell growth on bright microscopy (Fig. 4G), EdU^+^ proliferating cells decreased significantly as compared with cells transduced by lentivirus, but the five binding sites remained intact (Fig. 4, H and I). No difference was detected on the number of apoptotic cells (not shown). The PMP22 expression increased in Δ*Runx* cells in immunofluorescence staining. However, the PMP22 protein expression was retained in perinuclear-reticular structures, especially in Golgi body (not shown).

### RUNX1/3 regulates *Pmp22* promoter usage

**Fig. 5:**
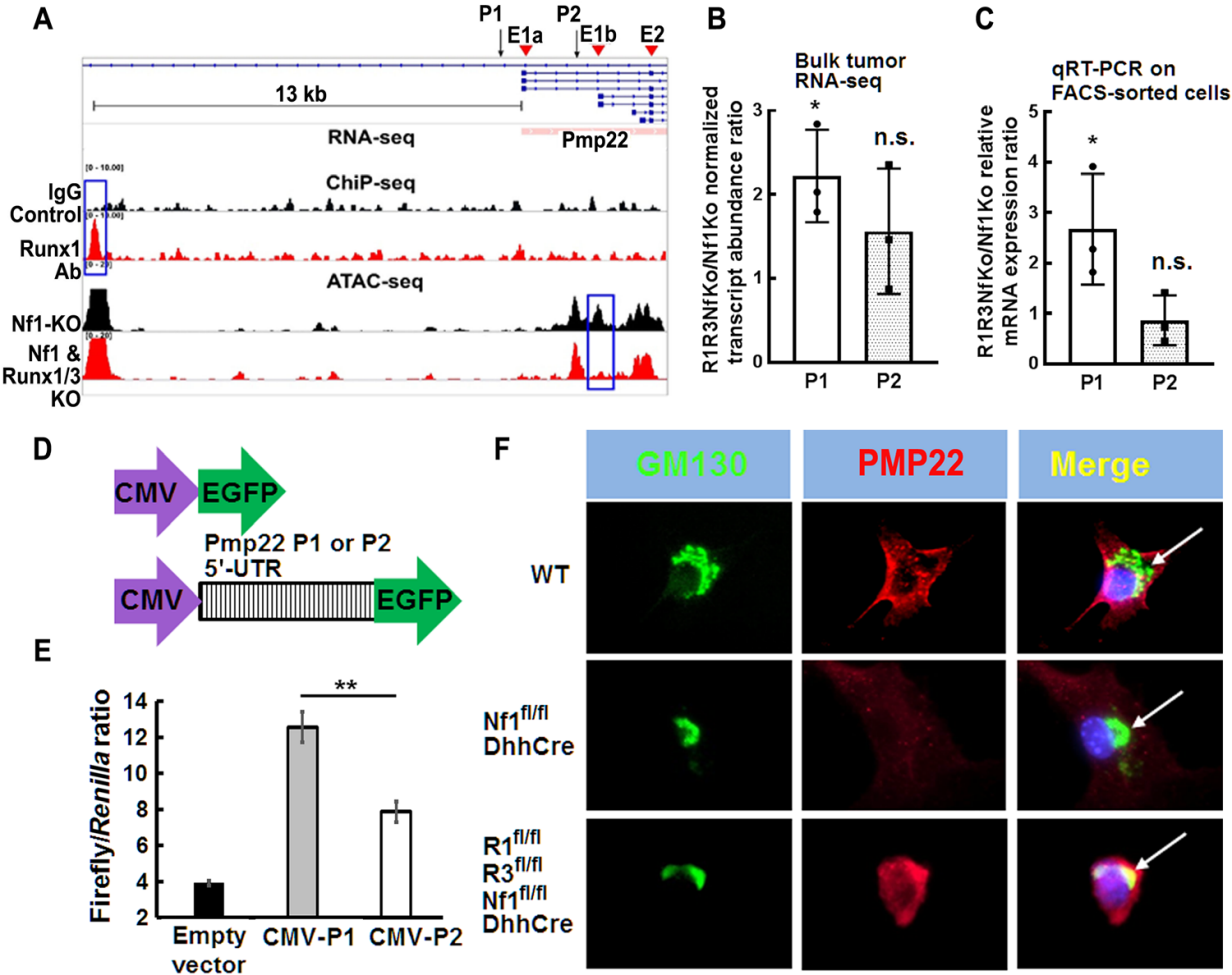
*Runx1/3* regulates the alternative promoter usage and affects posttranslational modification of *Pmp22*. (**A**) Upper two bands: RUNX1 ChIP-seq showed the binding peak near *Pmp22* (blue box). IgG was used as control. Lower two bands: ATAC-seq on *Nf1^fl/fl^;DhhCre* and *Runx*1*^fl/fl^;Runx3^fl/fl^;Nf1^fl/fl^;DhhCre* showed more *Pmp22* P2 open chromatin in *Nf1^fl/fl^;DhhCre* SCs (blue box). Top-right corner arrow pointed to TSS. Underneath, red bars indicated that *Pmp22* mRNA expression was up-regulated in *Runx*1*^fl/fl^;Runx3^fl/fl^;Nf1^fl/fl^;DhhCre* versus *Nf1^fl/fl^;DhhCre* mice. Ab, antibody. (**B**) Ratio of normalized *Pmp22* P1 (white bar) and P2 (light gray bar) transcript abundance in *Runx*1*^fl/fl^;Runx3^fl/fl^;Nf1^fl/fl^;DhhCre* (R1R3Nf1Ko) versus *Nf1^fl/fl^;DhhCre* (Nf1Ko) from bulk tumor RNA-seq data. (**C**) qRT-PCR showed *Pmp22* P1 (white bar) and P2 (light gray bar) relative mRNA expression in *Runx1^fl/fl^;Runx3^fl/fl^;Nf1^fl/fl^;DhhCre* (R1R3Nf1Ko) over *Nf1^fl/fl^;DhhCre* (Nf1Ko) from FACS-sorted EGFP^+^ cells. n.s., not significant. (**D**) Diagram showed the cloning strategy of Pmp22 P1 5′-UTR or P2 5′-UTR with CMV into pGL2 luciferase reporter assay vector. Only CMV was used as control. (**E**) Luciferase reporter assay showed that P2 5′-UTR (CMV-P2) had less posttranscriptional modification activity compared with P1 5′-UTR (CMV-P1) in immortalized WT mouse SCs. (**F**) Representative immunofluorescence images of GM130 (green) and PMP22 (red) in WT (top), *Nf1^fl/fl^;DhhCre* (middle), and *Runx*1*^fl/fl^;Runx3^fl/fl^;Nf1^fl/fl^;DhhCre* (bottom) mouse neurofibroma/DRG SCs. White arrows pointed to GM130 (Golgi body) and PMP22 colocalization. *P < 0.05 and **P < 0.01.

*Pmp22* has four protein-coding transcript variants [ENSMUST00000108702 (encoding using P1 promoter), ENSMUST0000001836 (encoding using P2 promoter), ENSMUST00000108700, and ENSMUST00000108701] (www.ensembl.org). Although all four transcripts can produce the same protein product, the preferential usage of each transcript depends on tissue types and developmental context. Analysis of chromatin accessibility by ATAC-seq revealed a preponderance of open chromatin accessibility at P2 region in FACS-sorted *Nf1^fl/fl^;DhhCre* SCs compared with *Runx1^fl/fl^;Runx3^fl/fl^;Nf1^fl/fl^;DhhCre* tumor SCs (Fig. 5A). Notably, *Pmp22* mRNA relative expression fold change (Fig. 2E) was lower than bulk tumor RNA-seq analysis (2.3×; table S1). One of the possibilities is that different transcripts (promoters) might contribute to this difference because RNA-seq only identified statistically significant expressed transcripts, while the primers we used in the bulk tumor qRT-PCR recognized all four transcripts. To determine whether that was the case, we checked the abundance of each transcript from bulk tumor RNA-seq data using Kallisto. We found that the abundances of ENSMUST00000108700 and ENSMUST00000108701 were negligible. Only ENSMUST00000108702 and ENSMUST0000001836 were abundant. *Runx1^fl/fl^;Runx3^fl/fl^;Nf1^fl/fl^;DhhCre* P1 transcript was 2.3-fold up-regulated compared with *Nf1^fl/fl^;DhhCre* (*P* < 0.05; Fig. 5B). The P2 transcript was ~2× more abundant than the P1 transcript in both tumors, but no statistically significant differential expression was detected between; therefore, P2 transcript’s effect on the gene-level differential change could be small in the bulk tumor RNA-seq. To exclude the influence of *Pmp22* RNAs from WT SCs and possible other tissues in bulk tumors, we checked the above two transcript variants’ relative mRNA expression levels on FACS-sorted EGFP^+^ SCs using special primers that only recognized each transcript by qRT-PCR. Consistent with the RNA-seq data, we found that only P1 transcript showed statistically significant differential expression in *Runx1^fl/fl^;Runx3^fl/fl^;Nf1^fl/fl^;DhhCre* versus *Nf1^fl/fl^;DhhCre*, with a 2.7-fold up-regulation (*P* < 0.05; Fig. 5C). These results indicated that P1 transcript was the actual contributor on *Pmp22* gene expression in SCs in both genotypes, while in *Nf1^fl/fl^;DhhCre* tumor cells, P2 might have been used to drive Pmp22 expression in other tissues.

It is previously reported that PMP22 protein expression might be partially influenced by the conserved alternative 5′-UTR (*28*). RegRNA2 search (http://regrna2.mbc.nctu.edu.tw) showed that there was a significant difference in the motif compositions of P1 and P2 5′-UTR regions; therefore, they might affect posttranscriptional control and translation efficiency. Thus, we adapted a luciferase reporter assay system to test whether the 5′-UTRs of the *Pmp22* P1 and P2 transcripts affect the PMP22 translation in our system. The EGFP luciferase was activated by two components: a cytomegalovirus (CMV) promoter and the P1 5′-UTR or P2 5′-UTR (Fig. 5D). CMV alone caused low luciferase transcriptional activity but was markedly increased by P1 5′-UTR or P2 5′-UTR (Fig. 5E). Significantly higher luciferase activity was driven by the P1 5′-UTR promoter vector compared with the P2 5′-UTR promoter (Fig. 5E), suggesting that the P1 5′-UTR had stronger activity in modifying posttranscriptional Pmp22 mRNA compared with the P2 5′-UTR.

In WT SCs, PMP22 is rapidly degraded in the ER, so that only a small portion of the total PMP22 accumulates in the Golgi body, to be translocated to the SC membrane when axonal contact and myelination occur (*29*). We performed immunofluorescence staining of PMP22 together with a cis-Golgi marker, GM-130, to visualize the PMP22 localization. The PMP22 expression was high in WT mouse SCs, both on the cell membrane and in the Golgi body (fig. S5F), but was almost undetectable in *Nf1^fl/fl^;DhhCre* tumor cells. High expression of PMP22 was detected in the *Runx1^fl/fl^;Runx3^fl/fl^;Nf1^fl/fl^;DhhCre* tumor cells in intracellular structures, including Golgi body, suggesting that RUNX1/3 changes the intracellular trafficking of PMP22. Together, we concluded that the combined outcome of the *Runx1/3*-mediated *Pmp22* promoter usage and protein metabolism leads to alternative diminished *Pmp22* expression in *Nf1^−/−^* SCs and SCPs and neurofibromagenesis.

### Ro5-3335 decreases neurofibroma growth by inhibiting SC proliferation and inducing SC apoptosis

**Fig. 6:**
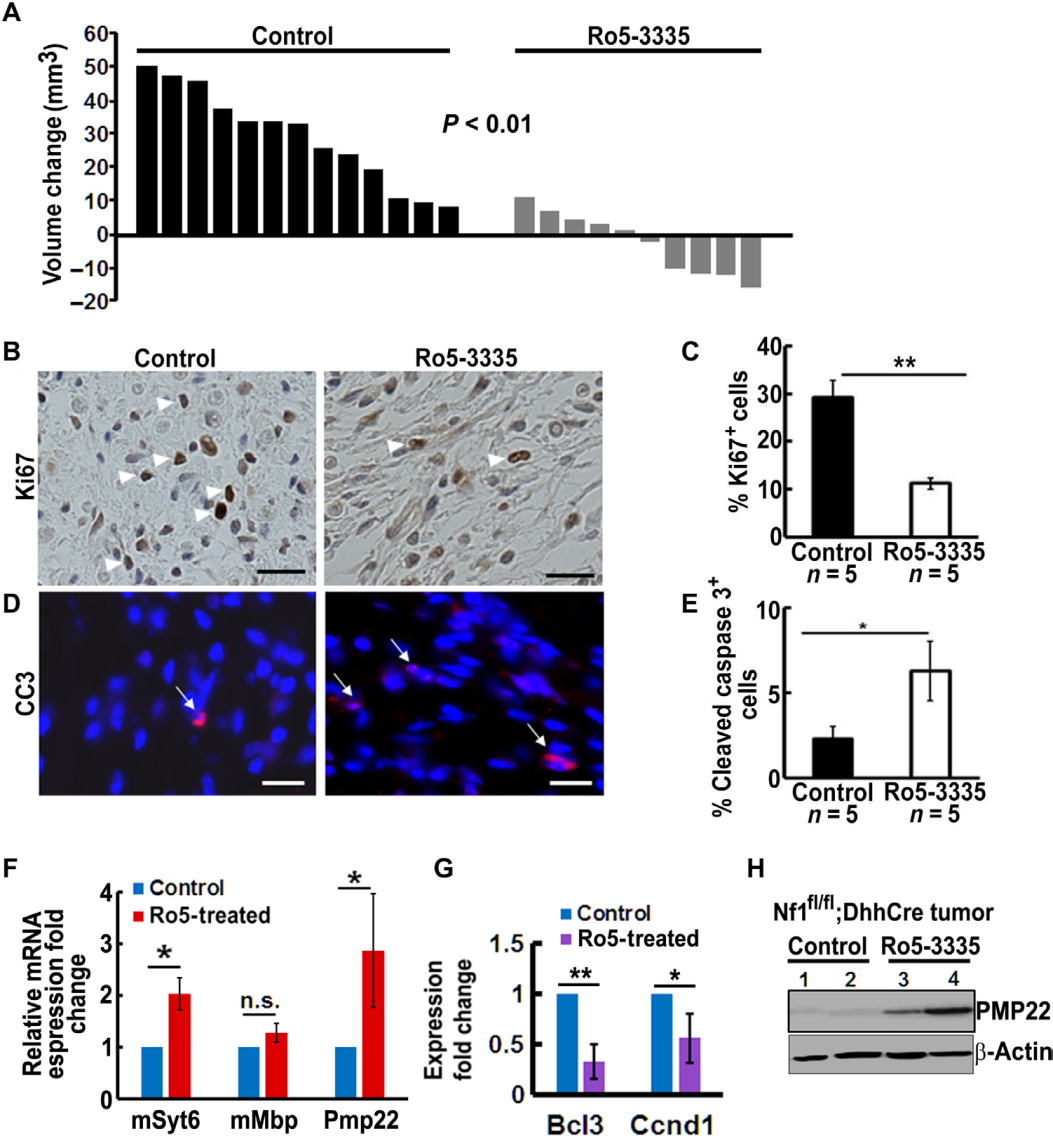
RUNX/CBFB interaction inhibitor, Ro5-3335, significantly decreases mouse neurofibroma growth in vivo. (**A**) Waterfall plot showed mouse tumor volume change. Data from each mouse were shown as a single bar. Change in tumor volume was quantified between 7 and 9 months for each individual mouse treated with vehicle control (control) (black bars; *n* = 13) or Ro5-3335 (gray bars; *n* = 10) for 8 weeks. (**B** and **C**) Ro5-3335–inhibited neurofibroma cell proliferation readout by the percentage of Ki67^+^ cells. (**D** and **E**) Ro5-3335–induced neurofibroma cell apoptosis as assessed by the percentage of CC3^+^ cells. (**F** and **G**) Relative mRNA expression of Ro5-3335–treated mouse neurofibromas compared with vehicle controls on up- (F) and down-regulated (G) genes. (**H**) Western blot of PMP22 in *Nf1^fl/fl^;DhhCre* tumors (1 and 2) and Ro5-3335–treated *Nf1^fl/fl^;DhhCre* tumors (3 and 4). β-Actin was used as loading control. *P < 0.05 and **P < 0.01.

Last, we tested in vivo the effect of inhibition of *RUNX/Cbf-*β activity using the small molecular inhibitor, Ro5-3335, which inactivates RUNX and *Cbf-*β functions by sandwich-like binding to RUNX and CBF-B, thereby interrupting the association between them (*30*). Because Ro5-3335 can be toxic, we first determined the maximum tolerated dose (MTD) and dose de-escalation. We defined the MTD as 100 mg/kg twice daily from Mondays to Fridays and once daily on Saturdays and Sundays. We then treated the *Nf1^fl/fl^;DhhCre* mice by oral gavage Ro5-3335 (*n* = 12) or vehicle control (*n* = 13) at MTD for 8 weeks. A few mice lost <10% weight during the first 2 weeks, but all regained normal weight thereafter. Two drug-treated mice were removed from the cohort because of severe dermatitis and were excluded from the statistical analysis. All other mice survived, and there were no obvious side effects apart from drowsiness due to Ro5-3335 benzodiazepine derivative effects (*30*). Volumetric measurement showed a significant decrease in tumor volume in Ro5-3335–treated mice compared with vehicle controls (Fig. 6A). Ro5-3335 significantly reduced the number of Ki67^+^ proliferating cells (Fig. 6, B and C) and induced CC3^+^ apoptotic cells (Fig. 6, D and E). The relative mRNA expression changes on four selected genes from RNA-seq (two up-regulated and two down-regulated in genetic results) were consistent with the genetic results (Fig. 6, F and G). Western blot verified that the PMP22 protein expression increased in these Ro5-3335–treated mouse neurofibromas compared with vehicle controls (Fig. 6H).

## DISCUSSION

In this study, we performed in-depth investigation into the cooperative oncogenic roles of *Runx1/3* in *Nf1* neurofibromagenesis. We showed that either genetic deletion or pharmacological inhibition of *Runx1/3* function decreased mouse neurofibroma burden in vivo. We further showed that the underlying mechanism was that *Runx1/3* regulated alternative promoter usage and markedly induced levels of protein expression of PMP22, a direct target of RUNX1/3, to drive neurofibromagenesis. The RUNX family of genes can play pivotal roles in the initiation and/or maintenance of tumor cells via transcriptional misregulation, DNA repair defects, and genomic instability and promotes the development of cancers (*12*). They often compensate each other upon conditional inactivation of one *Runx* gene. We showed that *Runx3* compensated *Runx1* to contribute to neurofibromagenesis but that dual deletion of *Runx1/3* in the *Nf1^fl/fl^;DhhCre* mice overcame the gene dose compensation and exhibited more durable effects on tumor initiation and/or maintenance. Notably, the *Runx1/3* double-knockout mice were still dying, although the mouse survival time was prolonged (Fig. 1A). It is unlikely that *Runx2* compensated the *Runx1/3* activities because RNA-seq results showed that the *Runx2* expression was 2.8-fold down in *Runx1^fl/fl^;Runx3^fl/fl^;Nf1^fl/fl^;DhhCre* versus *Nf1^fl/fl^;DhhCre* mouse tumors (not shown). Further experiments are needed to determine the genes/pathways that might be involved in the remaining tumors.

The RUNX transcription factors play important roles during the development in both the central and peripheral nervous systems. In the peripheral nervous system, RUNX1 and RUNX3 orchestrate dynamic gene expression change during the differentiation and maturation of DRG neurons (*31*). RUNX1 promotes adult mouse neurosphere proliferation and neuronal differentiation (*32*). We found that overexpressed *Runx1/3* represses *Pmp22* to contribute to neurofibroma initiation and maintenance. PMP22 is highly expressed in myelinating SCs and contributes to membrane organization in compact myelin to maintain the integrity of myelinated peripheral nerves (*19*). We show that *Pmp22* expression decreases significantly in *Nf1^−/−^* neurofibroma SCs and increases after *Runx1/3* knockout or RUNX1/3-*Pmp22*–binding site deletion in mouse neurofibroma SCs. This might contribute to the increase in neurofibroma nonmyelinating SCs, as compared with *Runx1/3* knockout nerves in electronic micrographs (not shown).

PMP22 expression has also been detected outside of myelinating SCs and can play important functions in cell proliferation, differentiation, and death. PMP22 protein is expressed in rat DRG SCs at embryonic day 16 (E16). These *PMP22*-expressing progenitors are multipotent and can generate non-neural cells (such as smooth muscle cells) in response to factors of the TGF-β family (*33*). We showed that the expression of *Pmp22* was increased in *Runx1^fl/fl^;Runx3^fl/fl^;Nf1^fl/fl^;DhhCre* mouse DRG/tumor spheres and that decreasing *Pmp22* expression elevated precursor number, as evidenced by increased sphere numbers (Fig. 3C). It is plausible that overexpression of Runx1/3 leads to low levels of PMP22 protein expression; therefore, P2 is used more to drive PMP22 protein expression in other tissues during neurofibroma formation. This hypothesis is strongly supported by ATAC-seq data on FACS-sorted EGFP^+^ tumor SCs, in which there is more open chromatin access in neuronal P2 promoter in *Nf1^fl/fl^;DhhCre* cells (Fig. 5A), RNA-seq data on bulk tumor (Fig. 5B), and qRT-PCR results on FACS-sorted EGFP^+^ tumor SCs (Fig. 5C).

Our results also suggest that the reduction of PMP22 proteins in *Nf1^fl/fl^;DhhCre* mouse neurofibromas may be attributed not only to reduced transcriptional regulation but also possibly to loss of RNA stability and diminished rate of posttranscriptional and/or translational modification in neurofibroma SCs, and further experiments are needed to determine whether and how these posttranscriptional steps are involved in regulating PMP22 protein expression. PMP22 expression in neurofibroma may result in alternative availability of the glial PMP22 protein during the cell cycle, leading to aberrant regulation of cell growth control during neurofibromagenesis. On the other hand, *Pmp22* mRNA levels are not always consistent with PMP22 protein expression. The varied expression of PMP22 in different tumor types indicates the role of PMP22 in growth arrest, and differentiation may be cell and tissue specific. PMP22 mRNA levels in colon cancer are not altered compared with normal tissue, yet protein levels, as measured by IHC and Western blot analysis, are significantly reduced in colon carcinoma samples compared with normal tissue (*34*). However, the rather subtle induction of *Pmp22* RNA and use of alternative promoters would not explain the markedly induced levels of PMP22 protein upon elimination of RUNX factors in our system. There are several possible explanations: (i) Different promoters might be used during neurofibroma development; (ii) transcription and posttranscriptional modification might be involved; (iii) translation might play an important role; and (iv) it might be PMP22 protein accumulation due to increased myelin sheaths (Fig. 1D), which can stabilize PMP22 protein, in *Runx1^fl/fl^;Runx3^fl/fl^;Nf1^fl/fl^;DhhCre*. All these possibilities highlight the need for dynamic PMP22 protein analysis in neurofibroma SCs.

In normal SCs, TEAD1 and its coactivators are required for *PMP22* expression and for the expression of early growth response protein 2 (EGR2) (*27*). Enhancers that regulate *PMP22* expression are regulated by the SRY sex-determining region Y-box 10 (SOX10) and EGR2/KROX20 transcription factors (*27*). EGR2 and SOX10 activity mediates the developmental induction of *Pmp22* expression by histone modification (*35*). Contrary to activation, we show that RUNX1/3 represses *Pmp22* in *Nf1^−/−^* SCs. Although the predicted SOX10-binding motif [consensus: AATTCATG, using the CIS-BP (Catalog of Inferred Sequence Binding Preferences) database] is located in chr11:63115220-63115227 and the locations between RUNX1/3 peaks and SOX10 probably overlap, no SOX10-binding motifs have been identified between our RUNX1/3 peak and the first exon of *Pmp22*. SOX10 may compete with RUNX1/3 for the same binding site because protein-protein interaction analysis shows that RUNX1/3 and SOX10 are not direct interacting partners. SOX10 expression is down-regulated in *NF1^−/−^* neurofibromas (*36*), and it is possible that RUNX1/3 binds to this TF-binding motif in our system to drive neurofibromagenesis. However, the effect on both *Pmp22* and *Mbp* suggests that perhaps common regulators of *Mbp* and *Pmp22* may be involved during tumor initiation and/or progression. Notably, the RUNX1-binding peak is located 17.9 kb upstream of *Pmp22* TSS (Fig. 5A). This suggests that a chromatin looping structure might be involved in the binding process. Chromosome conformation capture experiments will help to better understand the mechanisms. Besides *Pmp22*, it is highly possible that other RUNX1/3 targets, such as SDC4, ERN1, and/or MBP, might be involved in tumor formation.

Targeting transcription factors is becoming a realistic option with increased understanding of transcription factor biology and technological advances. Increased expression of other RUNX family member compensation for the antitumor effect elicited by single RUNX member silencing suggests that simultaneous attenuation of all RUNX family members might lead to much stronger antitumor effects than suppression of individual RUNX members. Morita *et al*. (*37*) show that targeting RUNX clustering using pyrrole-imidazole polyamides bind to RUNX-binding consensus sites (5′-TGTGGT-3′ and 5′-TGCGGT-3′) is effectively against AML. During Ro5-3335 treatment, RUNX/CBFB-binding sites are blocked, and none of the RUNX can bind to CBFB to achieve their transcriptional activities. It is different from the *Runx1* genetically conditional knockout, in which RUNX1 has no function but CBFB function is undisturbed, and the compensated *Runx3* gene produces RUNX3 that can bind to CBFB to achieve RUNX transcriptional activities and contribute to neurofibroma formation.

In summary, we show that the SC myelinating gene *Pmp22* serves as a tumor suppressor to contribute to neurofibromagenesis. Overexpression of PMP22 in mouse neurofibroma SCs decreases cell proliferation. Loss of *Nf1* activates RUNX1/3 to enable tumor initiation by repressing *Pmp22* through alternative promoter usage and other mechanisms to induce protein level expression of PMP22. The efficacy of Ro5-3335 on mouse neurofibroma growth suggests that disruption of RUNX/CBFB interaction or targeting *RUNX* gene cluster might provide a novel therapeutic strategy for patients with neurofibroma.

## MATERIALS AND METHODS

### Animals

Mice were housed in temperature- and humidity-controlled facilities on 12-hour dark-light cycles with free access to food and water. The animal care and use committees of the Cincinnati Children’s Hospital Medical Center approved all animal procedures. Institutional Animal Care and Use Committee guidelines were followed with animal subjects. To study the specific function of SCs, we used *Dhh-Cre* transgenic mouse, where *Cre*-mediated recombination activity would result in deletion of the floxed Nf1 allele in SC/SCPs of the developing peripheral nerves at E12.5. We bred the *Runx1^fl/fl^;Runx3^fl/fl^* mice (*38*, *39*) onto the *Nf1^fl/fl^* background to obtain F1 generation (*Runx1^fl/+^;Runx3^fl/+^;Nf1^fl/+^*). We also bred the *Runx1^fl/fl^;Runx3^fl/fl^* mice with *Nf1^fl/+^*;*DhhCre^+^* mice to obtain *Runx1^fl/+^;Runx3^fl/+^;Nf1^fl/+^;DhhCre* mice. We interbred *Runx1^fl/+^;Runx3^fl/+^;Nf1^fl/+^* mice to obtain *Runx1^fl/fl^;Runx3^fl/fl^;Nf1^fl/fl^* mice. We then bred *Runx1^fl/fl^;Runx3^fl/fl^;Nf1^fl/fl^* mice with *Runx1^fl/+^;Runx3^fl/+^;Nf1^fl/+^;DhhCre* mice to obtain *Runx1^fl/fl^;Runx3^fl/fl^;Nf1^fl/fl^;DhhCre* mice. Littermates *Runx1^fl/+^;Runx3^fl/+^;Nf1^fl/fl^;DhhCre*, *Runx1^fl/+^;Runx3^fl/fl^;Nf1^fl/fl^;DhhCre*, *Runx1^fl/fl^;Runx3^fl/+^;Nf1^fl/fl^;DhhCre*, or *Runx1^fl/fl^;Runx3^fl/fl^;DhhCre* mice were used as controls. Genotyping was performed as described (*16*).

### Neurofibroma sphere formation

Mouse neurofibroma/DRG-derived sphere culture was performed as described (*26*). Specifically, we plated trypan blue–negative cells at 1 × 10^4^ cells per 24-well low-binding plates in 1 ml of medium containing Dulbecco’s modified Eagle’s medium (DMEM):F-12 (3:1) and recombinant human epidermal growth factor (20 ng/ml; R&D Systems), 20 ng/ml recombinant human basic fibroblast growth factor (R&D Systems), 1% B-27 (Invitrogen), and heparin (2 μg/ml; Sigma-Aldrich). We maintained cultures at 37°C and 5% CO_2_. To passage, we centrifuged sphere cultures, dissociated and replated at 1 × 10^4^ cells/ml in fresh sphere medium as described (*6*).

### Immunohistochemistry

Tissue was embedded in paraffin, and 6-μm sections were cut, stained with either H&E or toluidine blue, and incubated overnight at 4°C with the following antibodies: anti-S100β (Dako, Carpinteria, CA), Ki67 (Novocastra Leica Microsystems, Buffalo Grove, IL), anti-BrdU, or anti-CC3 (Cell Signaling Technology, Danvers, MA). Visualization methods were as described (*6*).

### Western blots

Western blots were performed using antibodies recognizing PMP22, β-tubulin, and β-actin (Cell Signaling Technology, Danvers, MA). At least three different tumor/cell lysates were analyzed per antigen.

### Tumorigenesis assay in nude mice

We subcutaneously injected 5 × 10^5^ mouse sphere cells per injection into athymic nude mice (males and females, Harlan, Indianapolis, IN). After 2 months, we dissected tumors and fixed them overnight in 4% paraformaldehyde for paraffin embedding or in Karnovsky fixative for electron microscopy.

### Lentiviral transduction

We transduced secondary *Nf1^fl/fl^;DhhCre* or *Runx1^fl/fl^;Runx3^fl/fl^;Nf1^fl/fl^;DhhCre* DRG/neurofibroma spheres with purified *shRNAs* or nontarget control lentivirus (Sigma-Aldrich, St. Louis, MO). We incubated lentiviral particles with neurofibroma spheres at multiplicity of infection of 1:10 to 1:50 for 4 days and counted sphere numbers. For in vivo transplantation, *Runx1^fl/fl^;Runx3^fl/fl^;Nf1^fl/fl^;DhhCre* DRG/neurofibroma spheres were transduced with lentivirus in the presence of Polybrene (8 μg/ml; Sigma-Aldrich) for 16 to 20 hours, followed by culturing for 4 days. Spheres were collected and dissociated into single cells for transplantation. For overexpression studies, we used puromycin at 1 μg/ml to eliminate untransduced cells.

### Microarray, RNA-seq, ChIP-seq, and ATAC-seq

The SuperSeries data of microarray (GSE122773), RNA-seq (GSE122774), ChIP-seq (GSE122775), and ATAC-seq (GSE122776) raw and processed data were deposited in Gene Expression Omnibus (www.ncbi.nlm.nih.gov/geo) as GSE122777. High-throughput sequencing was performed on the Illumina HiSeq 2500. We targeted 45 to 50 million reads per sample for RNA-seq and 25 to 30 million reads for ChIP-seq and ATAC-seq. Sequencing reads in FASTQ files were examined by FastQC (v0.11.5; www.bioinformatics.babraham.ac.uk/projects/fastqc).

For RNA-seq (*n* = 3 per genotype), total RNAs isolated from *Nf1^fl/fl^;DhhCre* and *Runx1^fl/fl^;Runx3^fl/fl^;Nf1^fl/fl^;DhhCre* mouse DRG/tumors were amplified using the Ovation RNA-Seq System v2 (NuGEN) according to the manufacturer’s protocol. The libraries were prepared with the Nextera XT DNA Sample Preparation Kit (Illumina Technologies). Paired-end reads were aligned to the mm10 reference genome using TopHat (v2.0.13). Aligned BAM files were converted into raw count files using feature counts (v1.4.6). The raw count tables were normalized using the Bioconductor’s edgeR-TMM normalization methods, and DEGs were detected using Bioconductor’s limma/voom package. The |fold change| > 2× and FDR *P* < 0.05 were used as cutoffs to detect DEGs. The abundance of each transcript variant was calculated using Kallisto (v0.43; https://pachterlab.github.io/kallisto).

For ChIP-seq (*n* = 1 per genotype), primary *Nf1^fl/fl^;DhhCre* mouse neurofibroma cells were used for the ChIP assay according to Magna ChIP instruction (Millipore, Billerica, MA) as described (*40*). Reads were trimmed using Trim Galore (v0.4.4; www.bioinformatics.babraham.ac.uk/projects/trim_galore) and aligned to the mouse reference genome (mm10) using Bowtie2 (v2.2.6; http://bowtie-bio.sourceforge.net/bowtie2). HOMER (v4.9; http://homer.ucsd.edu/homer) was used to predict and annotate differential peaks with the following parameters (-F 4 -L4 -c 2 -style factor -FDR 0.001). We considered differential peaks with FDR *P* < 0.001 as statistically significant.

ATAC-seq (*n* = 1 per genotype) was performed as described (*41*) using FACS-sorted EGFP^+^ *Nf1^fl/fl^;DhhCre* or *Runx1^fl/fl^;Runx3^fl/fl^;Nf1^fl/fl^;DhhCre* mouse DRG/tumor cells. Reads were trimmed using Trim Galore (v0.4.4) and aligned to the mm10 genome using Bowtie2 (v2.2.6). HOMER (v4.9) was used to predict and annotate differential peaks with the following parameters (−size 75 -mDist 50 -style factor -FDR 0.05). We considered differential peaks with FDR < 0.001 as statistically significant.

For microarray, tumor initiating–like cells were FACS-sorted from neurofibroma tumors, and total mRNA was extracted using the Qiagen Kit. Affymetrix Human Genome U133 Plus 2.0 array was used for transcriptome analysis. CEL files were preprocessed using Bioconductor’s affy package with Robust Multi-array Average normalization method. DEGs were predicted using Bioconductor’s limma package. The |fold change| > 2× and FDR *P* < 0.05 were used as cutoffs to detect DEGs.

### Luciferase reporter assay

CMV promoter sequence cloned from pCDNA3.1 (primers: forward, 5′-GACATTGATTATTGACTAG; reverse, 5′-TGGTGGAGCTCCCTGTAACTAGCTCTGCTTATATAGACC) was merged with mouse Pmp22 promoter region P1 5′-UTR or P2 5′-UTR, respectively. P1 and P2 were amplified from mouse genomic DNA using the following primers: P1 5′-UTR, 5′-AGCTCCACCAGAGAACCTCTCA-3′ (forward) and 5′-TGAGGAGTAGCAGTGTTGGACGG (reverse); P2 5′-UTR, 5′-TGACCCGCAGCACAGCTGTCTTTG-3′ (forward) and 5′-TGAGGAGTAGCAGTGTTGGACGG-3′ (reverse). PCR products were cloned into a pGL2 firefly luciferase reporter plasmid (Promega, Madison, WI) and verified by DNA sequencing. The putative mouse Pmp22 P1 5′-UTR and P2 5′-UTR with CMV were cloned into the pGL2 vector between Xba I and Fse I sites of the Dual-Luciferase Reporter Assay System vector (Promega), immediately downstream of the luciferase stop codon, according to the manual instructions. We used immortalized WT mouse SCs that are derived from the *Nf2^fl/fl^* mouse model for transfection (*42*). SCs at 60% confluence were cotransfected in triplicate using Lipofectamine with the abovementioned Dual-Luciferase Reporter Assay vectors P1, P2, or CMV only (control). CMV-*Renilla* was used as an internal control to normalize the transfection efficiency. Luciferase transcriptional activities were determined 48 hours after transfection using a dual-luciferase assay kit (Promega, Madison, WI).

### Runx-Pmp22–binding sites deletion by CRISPR-Cas9

We used the CRISPR-Cas9 design site crispr.mit.edu to identify guide RNAs (gRNAs) that target the putative Runx-Pmp22–binding region (Chr11 63114879-63115495). On the basis of efficiency and low frequency of off-target sites, we chose two gRNAs: mU6-driven gRNA 5′-AGTACAGGTTTCCCCCCTGG AGG-3′ and hU6-driven gRNA (reversely inserted into pLV vector) 5′-TGCGCATACAGAGTCCAACC AGG-3′, predicted to create a deletion of ~360 bps and result in deletion of five putative Runx-binding sites in Pmp22 gene. PCR products containing these two gRNAs were ligated into the pLV hUbC-Cas9-T2A-GFP backbone (plasmid #53190, Addgene, Cambridge, MA) after linearization using BsmB I (New England BioLabs, Ipswich, MA). This plasmid expressed the Cas9 protein, ligated two gRNAs, and GFP simultaneously (named pLV hUbC-Cas9-T2A-GFP-2gRNAs). Plasmids were sequenced to confirm proper insertion of gRNAs. Lentiviral particles were packaged as described (*43*). We incubated mouse neurofibroma SCs with pLV hUbC-Cas9-T2A-GFP-2gRNA lentivirus and Polybrene (8 μg/ml) for 16 hours and then changed to SC culture medium for 5 days. We FACS-sorted GFP high single cells at one cell per well to 96-well plates containing DMEM with 20% fetal bovine serum, β-heregulin (10 ng/ml) and forskolin (1 μM). Three to 4 weeks later, we screened clones by PCR. PCR primers are as follows: ACTAGACATGAAACGGTCTGC (forward) and GTTTATCCAGACCTGGCCATT (reverse). The anticipated PCR product lengths were 531 bps (WT) and 171 bps (knockout). Homozygous deletion was confirmed by sequencing on both directions.

### Statistics

We used a Gehan-Breslow-Wilcoxon log-rank test for Kaplan-Meier analysis. Neurofibroma growth was modeled by mixed-effects model analysis. *P* values were generated with a random-effects model analysis on log-transformed tumor volume data using the SAS mixed procedure (*44*). Fisher’s exact test was used on transplanted nude mice tumor formation analysis. We used unpaired two-tailed Student’s *t* tests to analyze the significance of cell proliferation and cell death quantification in tissue sections when two samples were compared. Data were reported as means ± SEM. *P* < 0.05 was considered significant.

## Supplementary Material

http://advances.sciencemag.org/cgi/content/full/5/4/eaau8389/DC1

Download PDF

Table S1

## SUPPLEMENTARY MATERIALS

Supplementary material for this article is available at http://advances.sciencemag.org/cgi/content/full/5/4/eaau8389/DC1 Fig. S1. Conditional knockout of Runx1 induces Runx3 overexpression in the Runx1fl/fl;Nf1fl/fl;DhhCre mouse neurofibromas. Fig. S2. Runx1/Runx3 drive neurofibromagenesis by activating oncogenic pathways and reprogramming the neuronal and immune systems. Fig. S3. ChIP-seq and ATAC-seq revealed the potential targets of Runx. Fig. S4. Gene expression of SC differentiation/myelination markers and RUNX family genes from existing transcriptomic data. Fig. S5. Gene expression of other known Pmp22 regulator in RNA-seq. Fig. S6. CRISPR-Cas9 approach deletes five putative Runx-binding sites in Pmp22 gene. Table S1. Differential gene level expression change in Runx1fl/fl;Runx3fl/fl;Nf1fl/fl;DhhCre mouse tumors versus Nf1fl/fl;DhhCre mouse tumors (FDR *P* < 0.05, |fold change| > 2×).
