# Supplementary material for: RUNX represses Pmp22 to drive neurofibromagenesis
Source: Sci Adv. 2019 Apr 24;5(4):eaau8389. doi: 10.1126/sciadv.aau8389 (PMC6482019; doi:10.1126/sciadv.aau8389)
Supplement: http://advances.sciencemag.org/cgi/content/full/5/4/eaau8389/DC1 [file supp_5_4_eaau8389__index.html]

Science Advances | Science Advances

## Supplementary Materials

**The PDF file includes:**

- Fig. S1. Conditional knockout of Runx1 induces Runx3 overexpression in the Runx1fl/fl;Nf1fl/fl;DhhCre mouse neurofibromas.
- Fig. S2. Runx1/Runx3 drive neurofibromagenesis by activating oncogenic pathways and reprogramming the neuronal and immune systems.
- Fig. S3. ChIP-seq and ATAC-seq revealed the potential targets of Runx.
- Fig. S4. Gene expression of SC differentiation/myelination markers and RUNX family genes from existing transcriptomic data.
- Fig. S5. Gene expression of other known Pmp22 regulator in RNA-seq.
- Fig. S6. CRISPR-Cas9 approach deletes five putative Runx-binding sites in Pmp22 gene.
- Legend for table S1

Download PDF

**Other Supplementary Material for this manuscript includes the following:**

- Table S1 (Microsoft Excel format). Differential gene level expression change in Runx1fl/fl;Runx3fl/fl;Nf1fl/fl;DhhCre mouse tumors versus Nf1fl/fl;DhhCre mouse tumors (FDR *P* < 0.05, |fold change| > 2×).

**Files in this Data Supplement:**

- Adobe PDF - aau8389\_SM.pdf
