# Supplementary material for: RUNX represses Pmp22 to drive neurofibromagenesis
Source: Sci Adv. 2019 Apr 24;5(4):eaau8389. doi: 10.1126/sciadv.aau8389 (PMC6482019; doi:10.1126/sciadv.aau8389)
Supplement: Download PDF [file aau8389_SM.pdf]

Science Advances
AAAS

advances.sciencemag.org/cgi/content/full/5/4/eaau8389/DC1

Supplementary Materials for

RUNX represses *Pmp22* to drive neurofibromagenesis

Ashley Hall, Kwangmin Choi, Wei Liu, Jonathan Rose, Chuntao Zhao, Yanan Yu, Youjin Na, Yuqi Cai, Robert A. Coover, Yi Lin, Eva Dombi, MiOk Kim, Ditsa Levanon, Yoram Groner, Elisa Boscolo, Dao Pan, P. Paul Liu, Q. Richard Lu, Nancy Ratner, Gang Huang, Jianqiang Wu*

*Corresponding author. Email: jianqiang.wu@cchmc.org

Published 24 April 2019, *Sci. Adv.* **5**, eaau8389 (2019)
DOI: 10.1126/sciadv.aau8389

The PDF file includes:

Fig. S1. Conditional knockout of Runx1 induces Runx3 overexpression in the Runx1fl/fl;Nf1fl/fl;DhhCre mouse neurofibromas.
Fig. S2. Runx1/Runx3 drive neurofibromagenesis by activating oncogenic pathways and reprogramming the neuronal and immune systems.
Fig. S3. ChIP-seq and ATAC-seq revealed the potential targets of Runx.
Fig. S4. Gene expression of SC differentiation/myelination markers and RUNX family genes from existing transcriptomic data.
Fig. S5. Gene expression of other known Pmp22 regulator in RNA-seq.
Fig. S6. CRISPR-Cas9 approach deletes five putative Runx-binding sites in Pmp22 gene.
Legend for table S1

Other Supplementary Material for this manuscript includes the following:

(available at advances.sciencemag.org/cgi/content/full/5/4/eaau8389/DC1)

Table S1 (Microsoft Excel format). Differential gene level expression change in Runx1fl/fl;Runx3fl/fl;Nf1fl/fl;DhhCre mouse tumors versus Nf1fl/fl;DhhCre mouse tumors (FDR $P < 0.05$, |fold change| > 2×).

Supplemental information

RUNX represses *Pmp22* to drive neurofibromagenesis

Figure S1

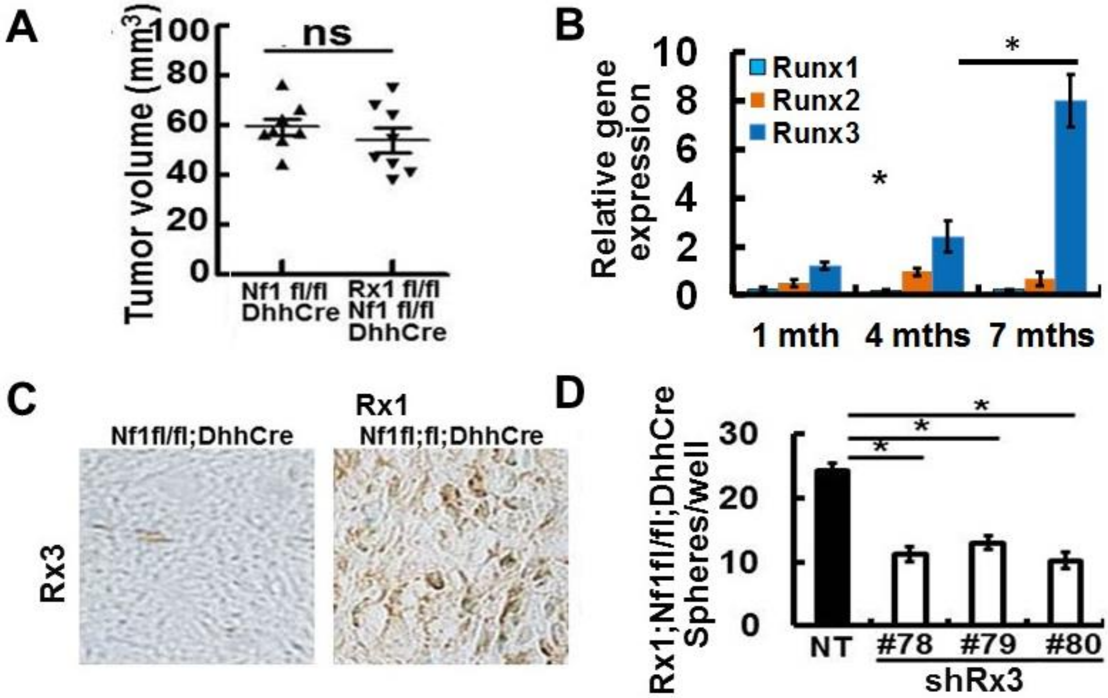

Fig. S1. Conditional knockout of Runx1 induces Runx3 overexpression in the Runx1fl/fl;Nf1fl/fl;DhhCre mouse neurofibromas. (A). Volumetric measurements on 7 months old *Runx1$^{fl/fl}$;Nf1$^{fl/fl}$;DhhCre* and *Nf1$^{fl/fl}$;DhhCre* mice. (**B)**. qRT-PCR showed Runx3 expression increases with time in *Runx1$^{fl/fl}$;Nf1$^{fl/fl}$;DhhCre* compared to *Nf1$^{fl/fl}$;DhhCre* mouse neurofibromas. (**C**) immunostaining of Runx3 in *Nf1$^{fl/fl}$;DhhCre* (left) and *Runx1$^{fl/fl}$;Nf1$^{fl/fl}$;DhhCre* (right) mouse neurofibromas. (**D**). All three shRunx3 clones significantly decrease 7 months Runx1$^{fl/fl}$*;Nf1$^{fl/fl}$;DhhCre* mouse neurofibroma sphere numbers compared to non-target control (NT).

Figure S2

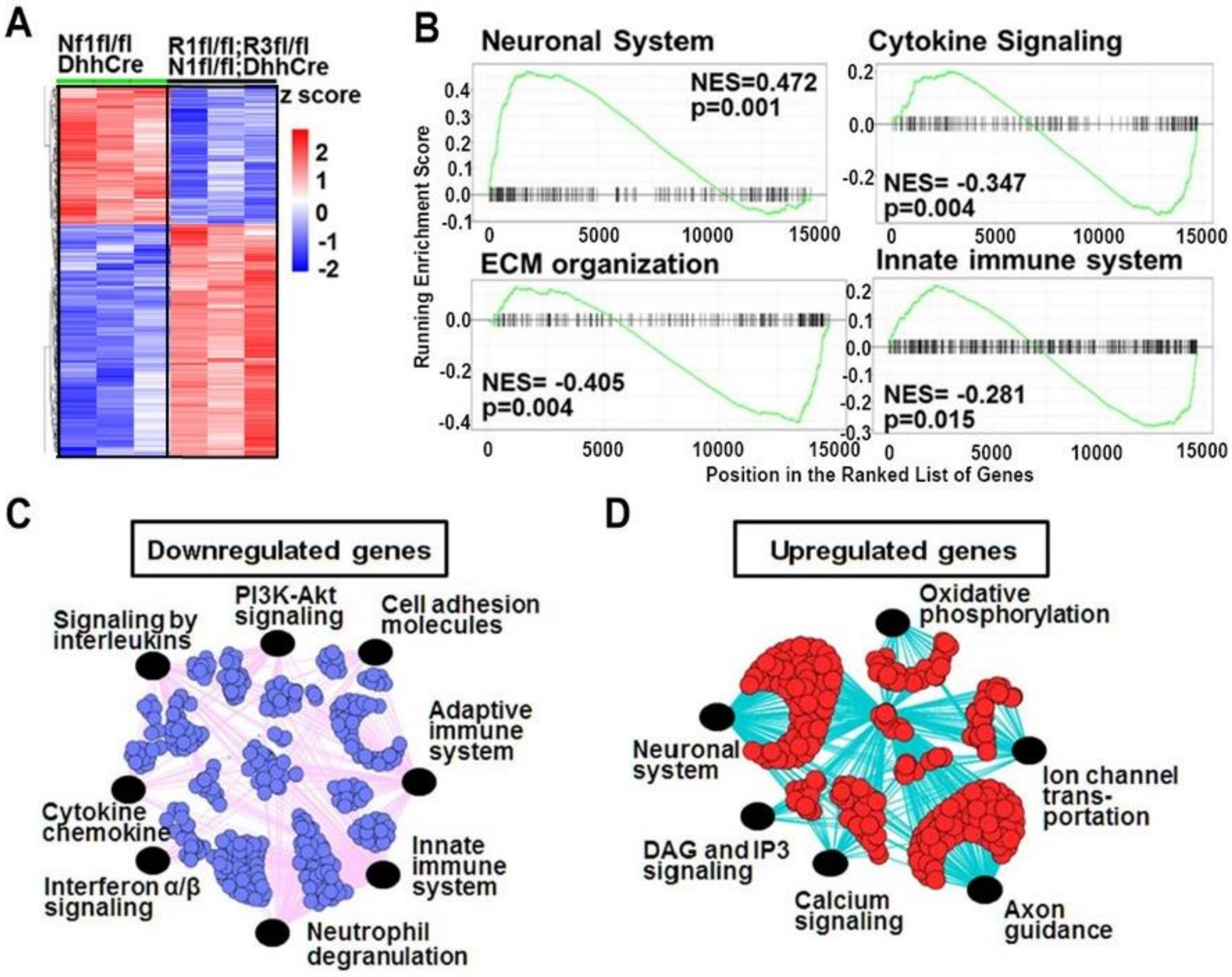

Fig. S2. Runx1/Runx3 drive neurofibromagenesis by activating oncogenic pathways and reprogramming the neuronal and immune systems. (A) Heatmap showed differentially expressed genes in Runx1$^{fl/fl}$;*Runx3*$^{fl/fl}$;*Nf1*$^{fl/fl}$;*DhhCre* (n=3) compared to *Nf1*$^{fl/fl}$;*DhhCre* mouse tumors (n=3). (**B**) GSEA enrichment analysis of RNA-seq data by comparing Runx1$^{fl/fl}$;*Runx3*$^{fl/fl}$;*Nf1*$^{fl/fl}$;*DhhCre* (n=3) to *Nf1*$^{fl/fl}$;*DhhCre*. Gene sets with FDR q values < 0.25 are plotted as a function of normalized enrichment scores (NES). (**C**) ToppCluster analysis showed top 10 functional networks (3 networks are similar or the same) among the genes and their associated biological processes downregulated in Runx1$^{fl/fl}$;*Runx3*$^{fl/fl}$;*Nf1*$^{fl/fl}$;*DhhCre* mouse

tumors . (**D**)ToppCluster analysis showed top 10 functional networks (5 networks are similar or the same) among the genes and their associated biological processes upregulated in Runx1$^{fl/fl}$;*Runx3*$^{fl/fl}$;*Nf1*$^{fl/fl}$;*DhhCre* mouse tumors.

Figure S3

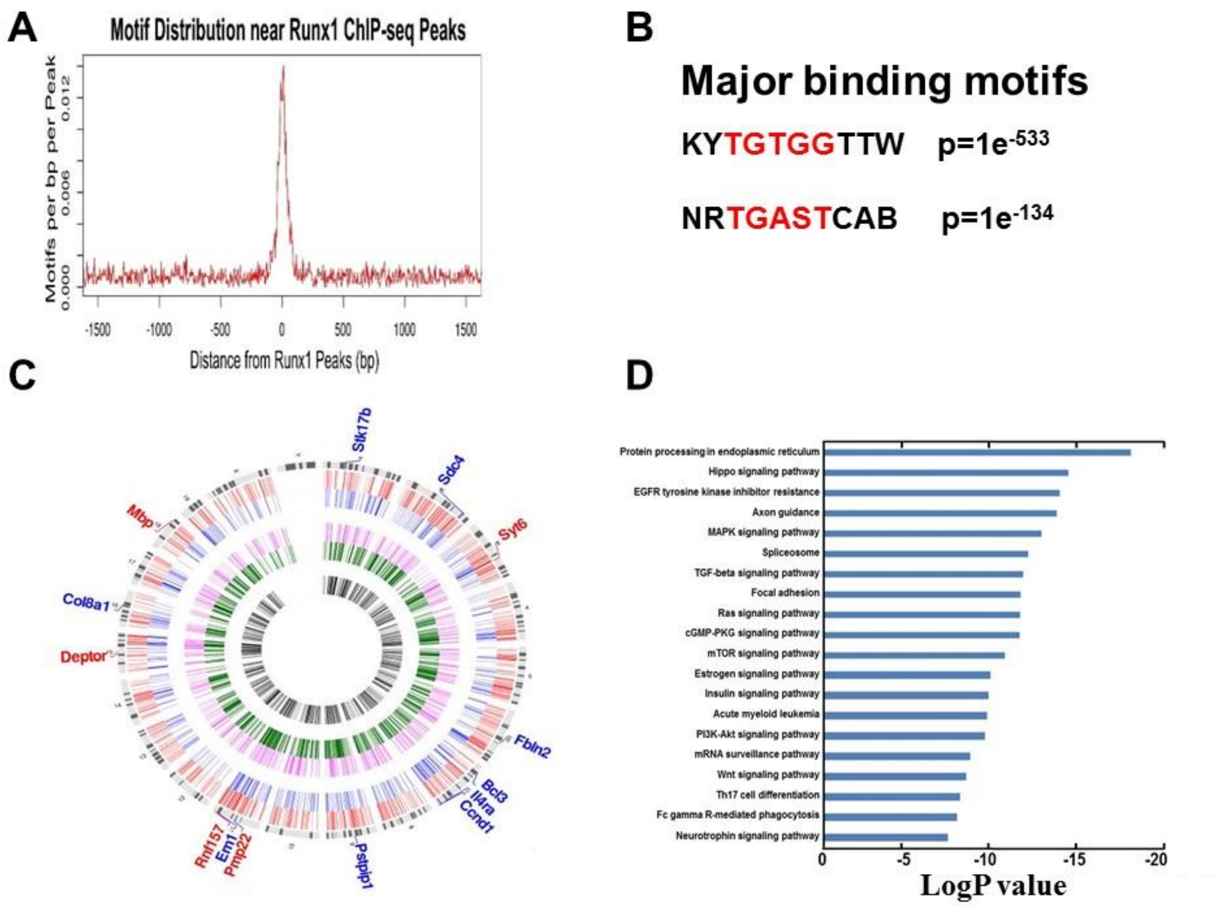

Fig. S3. ChIP-seq and ATAC-seq revealed the potential targets of Runx. (A) Motif distribution near Runx1 CHIP-seq peaks. A sharp peak centered at position zero surrounded by a larger conserved regulatory region of increased sequence up to 300 bp was observed. **(B)** De-novo transcriptional factor (TF) enrichment results showed two most enriched Runx1 TF motifs. (**C**) Combined RNA-seq (outside two circles, red and blue)), ATAC-seq (middle two circles, orange and green)), CHIP-seq (center circle, black). The 14 genes were listed on each chromosome (outside), red: upregulation, blue: down-regulation. (**D**) KEGG pathway by sorting of differential ATAC-seq peaks revealed areas of significances as highly enriched in protein processing in endoplasmic reticulum.

Figure S4

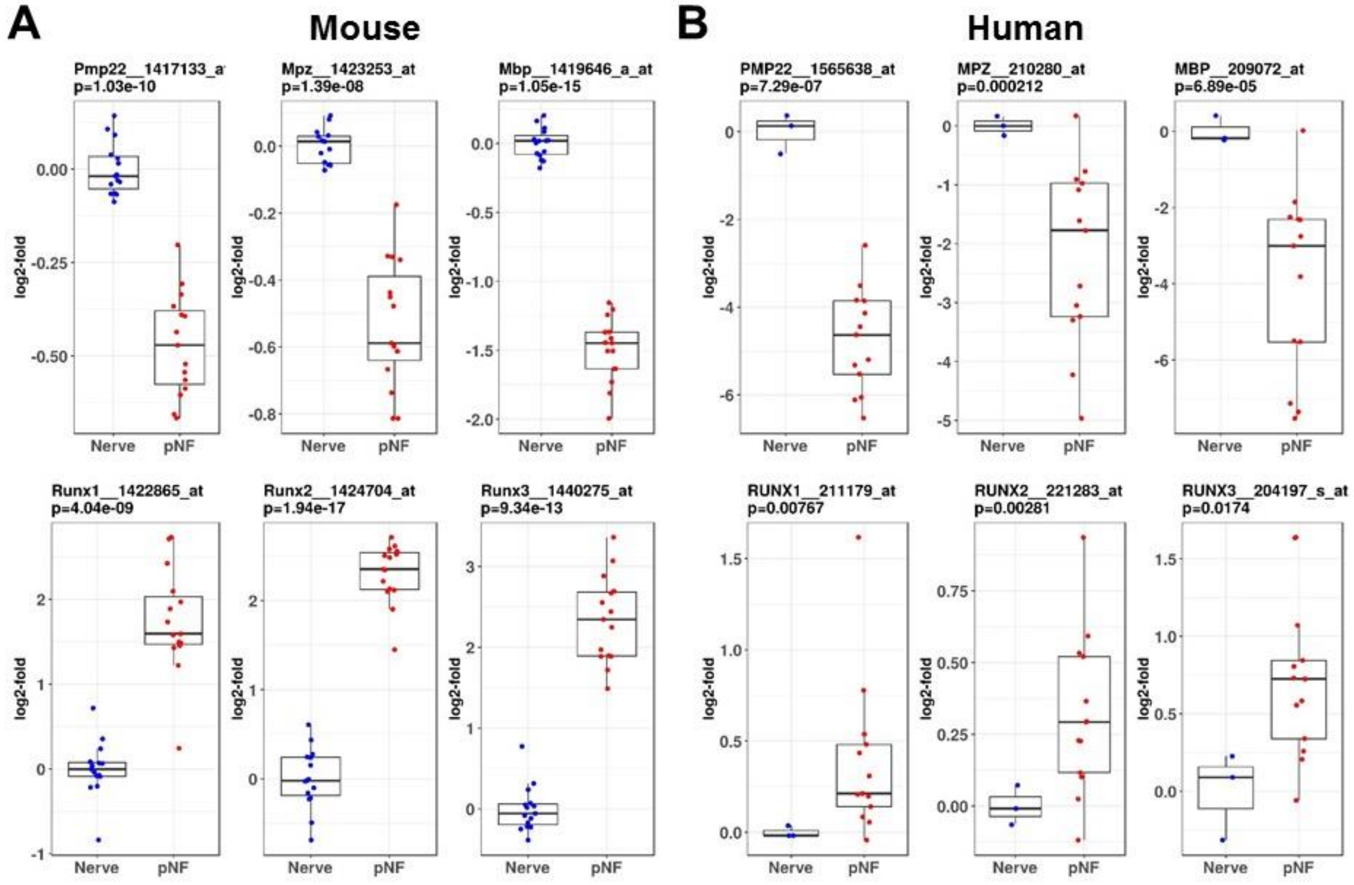

Fig. S4. Gene expression of SC differentiation/myelination markers and RUNX family genes from existing transcriptomic data. (A). Box and whisker plots show relative mRNA expression on Schwann cell differentiation/myelination markers (*Pmp22*, *Mpz* and *Mbp*) and Runx family genes (*Runx1*, *Runx2*, and *Runx3*) in mouse plexiform neurofibromas versus wild type nerve controls from GSE41747. (**B)**. Box and whisker plots show relative mRNA expression on Schwann cell differentiation/myelination markers (*PMP22, MPZ* and *MBP*) and RUNX family genes (*RUNX1*, *RUNX2*, and *RUNX3*) in human plexiform neurofibromas versus wild type nerve controls from GSE14038.

Figure S5

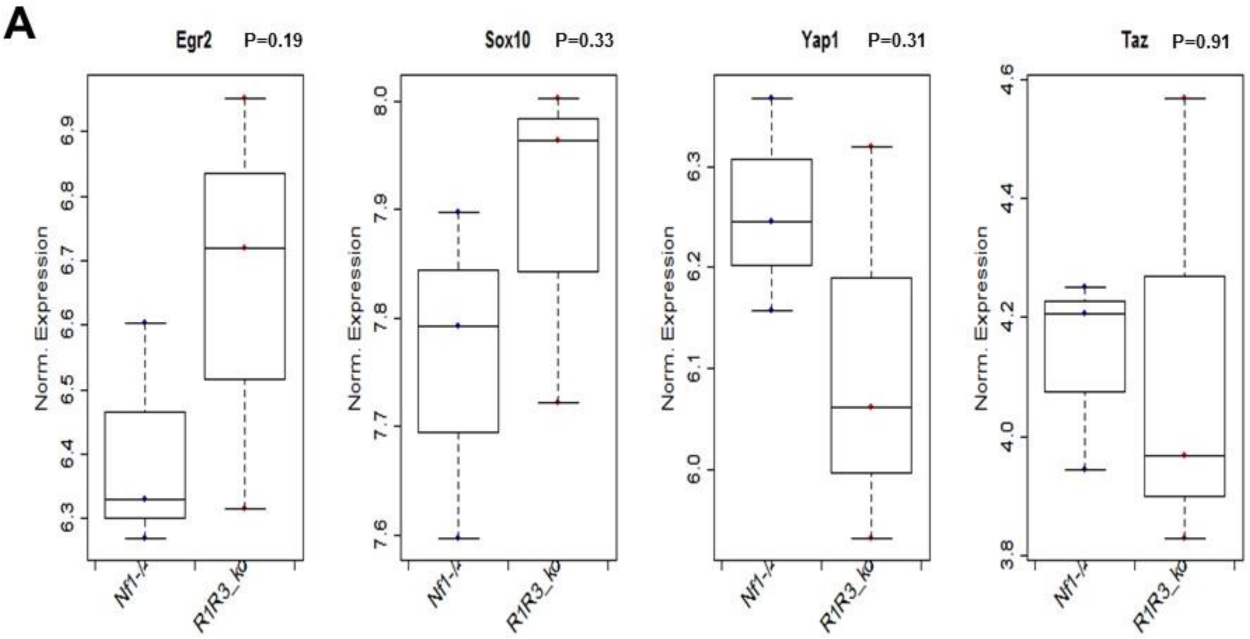

Fig. S5. Gene expression of other known Pmp22 regulator in RNA-seq. (A). Box and whisker plots show relative mRNA expression from *Runx1*$^{fl/fl}$*;Runx3*$^{fl/fl}$*;Nf1*$^{fl/fl}$*;DhhCre* mouse tumors (normalized to *Nf1*$^{fl/fl}$*;DhhCre* mouse tumors).

Figure S5

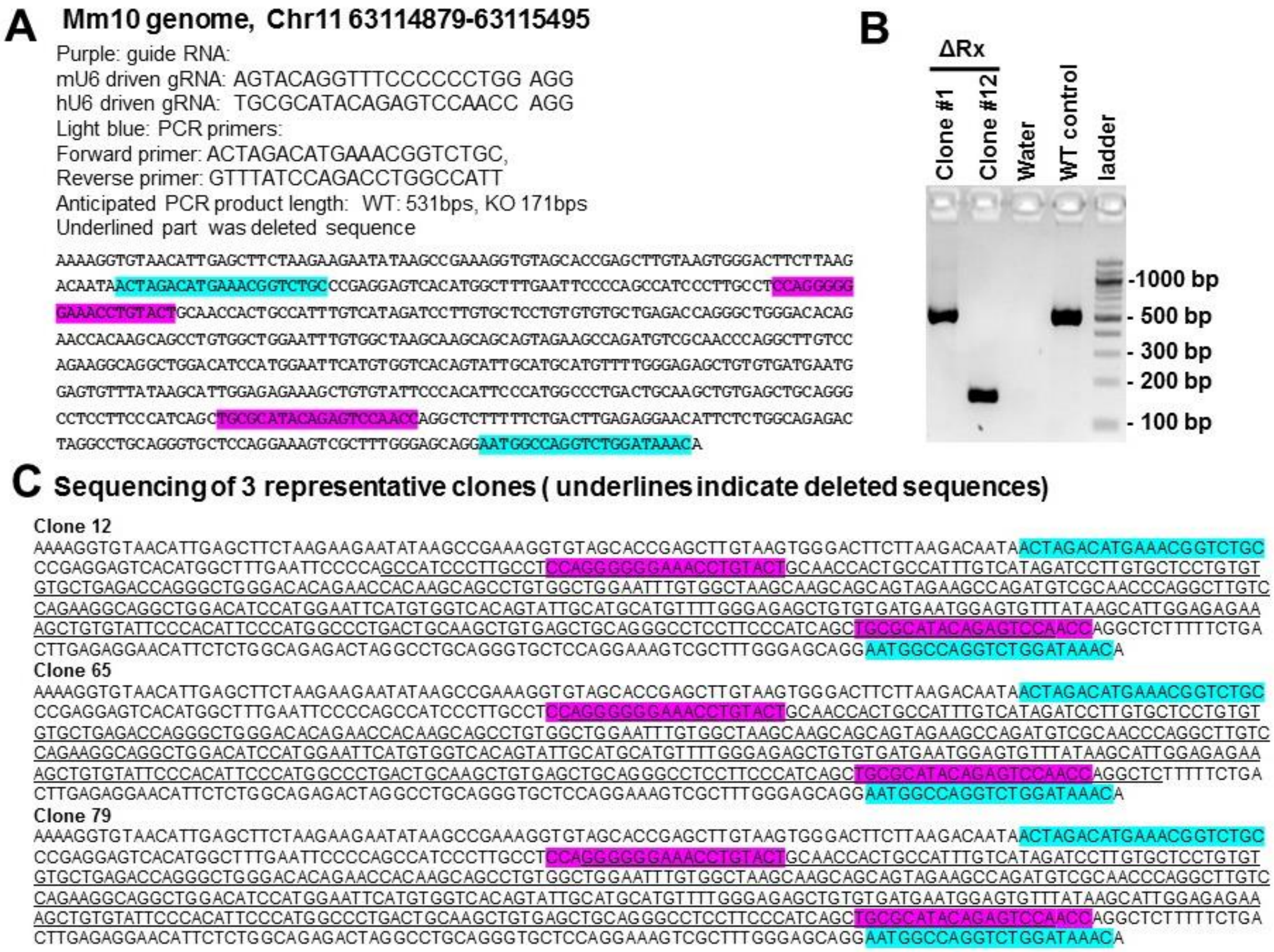

Fig. S6. CRISPR-Cas9 approach deletes five putative Runx-binding sites in Pmp22 gene.

(A). Scheme of CRISPR-Cas9 approach. List sequence containing 5 putative Runx binding sites in Pmp22 gene from Mm10 genome, Chr11 63114879-63115495. Purple: guide RNA: mU6 driven gRNA: AGTACAGGTTTCCCCCTGG AGG and

hU6 driven gRNA: TGCGCATACAGAGTCCAACC AGG

Blue: PCR primers: Forward primer: ACTAGACATGAAACGGTCTGC, Reverse primer: GTTTATCCAGACCTGGCCATT. Underlined part indicating deleted sequence. (**B**) Representative PCR gene imagine of PCR products. Anticipated PCR product length: WT:

531bps, KO 171bps. WT control DNA was from *Nf1*$^{fl/fl}$ mouse. (**C**) Sequences of 3 independent clones. Underline indicates homozygous deletion of each clone.

Table S1. Differential gene level expression change in Runx1fl/fl;Runx3fl/fl;Nf1fl/fl;DhhCre mouse tumors versus Nf1fl/fl;DhhCre mouse tumors (FDR *P* < 0.05, |fold change| > 2×). Blue: downregulation genes, Red: Upregulation genes.
